# Immune homeostasis and regulation of the interferon pathway require myeloid-derived Regnase-3

**DOI:** 10.1084/jem.20181762

**Published:** 2019-05-24

**Authors:** Matthias von Gamm, Annalisa Schaub, Alisha N. Jones, Christine Wolf, Gesine Behrens, Johannes Lichti, Katharina Essig, Anna Macht, Joachim Pircher, Andreas Ehrlich, Kathrin Davari, Dhruv Chauhan, Benjamin Busch, Wolfgang Wurst, Regina Feederle, Annette Feuchtinger, Matthias H. Tschöp, Caroline C. Friedel, Stefanie M. Hauck, Michael Sattler, Arie Geerlof, Veit Hornung, Vigo Heissmeyer, Christian Schulz, Mathias Heikenwalder, Elke Glasmacher

**Affiliations:** 1 Institute for Diabetes and Obesity, Helmholtz Diabetes Center at Helmholtz Zentrum München, German Research Center for Environmental Health, Neuherberg, Germany; 2 Institute of Molecular Toxicology and Pharmacology, Helmholtz Zentrum München, German Research Center for Environmental Health, Neuherberg, Germany; 3 Institute of Structural Biology, Helmholtz Zentrum München, German Research Center for Environmental Health, Neuherberg, Germany; 4 Center for Integrated Protein Science Munich, Chemistry Department, Technical University of Munich, Garching, Germany; 5 Institute of Environmental Medicine, Helmholtz Zentrum München, German Research Center for Environmental Health, Neuherberg, Germany; 6 Institute for Immunology, Biomedical Center, Ludwig-Maximilians-Universität München, Planegg-Martinsried, Germany; 7 Roche Pharma Research and Early Development, Large Molecule Research, Roche Innovation Center Munich, Penzberg, Germany; 8 Medizinische Klinik und Poliklinik I, Klinikum der Universität München, Ludwig-Maximilians-University, Munich, Germany; 9 Medigene Immunotherapies, Planegg-Martinsried, Germany; 10 Gene Center and Department of Biochemistry, Ludwig-Maximilians-Universität München, Munich, Germany; 11 Max von Pettenkofer-Institut für Hygiene und Medizinische Mikrobiologie, Ludwig-Maximilians-Universität München, Munich, Germany; 12 Institute of Developmental Genetics, Helmholtz Zentrum München, Munich, Germany; 13 Technische Universität München-Weihenstephan, Neuherberg-Munich, Germany; 14 German Center for Neurodegenerative Diseases, Munich, Germany; 15 Munich Cluster for Systems Neurology, Munich, Germany; 16 Monoclonal Antibody Core Facility, Institute for Diabetes and Obesity, Helmholtz Zentrum München, German Research Center for Environmental Health, Neuherberg, Germany; 17 Research Unit Analytical Pathology, Helmholtz Zentrum München, German Research Center for Environmental Health, Neuherberg, Germany; 18 Division of Metabolic Diseases, Department of Medicine, Technische Universität München, Munich, Germany; 19 Institute for Informatics, Ludwig-Maximilians-Universität München, Munich, Germany; 20 Research Unit Protein Science, Helmholtz Zentrum München, German Research Center for Environmental Health, Neuherberg, Germany; 21 Research Unit Molecular Immune Regulation, Helmholtz Zentrum München, German Research Center for Environmental Health, Munich, Germany; 22 German Center for Cardiovascular Research, partner site Munich Heart Alliance, Munich, Germany; 23 Division of Chronic Inflammation and Cancer (F180), German Cancer Research Center, Heidelberg, Germany

## Abstract

The RNase Regnase-1 is a master RNA regulator in macrophages and T cells that degrades cellular and viral RNA upon NF-κB signaling. The roles of its family members, however, remain largely unknown. Here, we analyzed *Regnase-3*–deficient mice, which develop hypertrophic lymph nodes. We used various mice with immune cell–specific deletions of *Regnase-3* to demonstrate that Regnase-3 acts specifically within myeloid cells. *Regnase-3* deficiency systemically increased IFN signaling, which increased the proportion of immature B and innate immune cells, and suppressed follicle and germinal center formation. Expression analysis revealed that Regnase-3 and Regnase-1 share protein degradation pathways. Unlike Regnase-1, Regnase-3 expression is high specifically in macrophages and is transcriptionally controlled by IFN signaling. Although direct targets in macrophages remain unknown, Regnase-3 can bind, degrade, and regulate mRNAs, such as *Zc3h12a* (*Regnase-1*), in vitro. These data indicate that Regnase-3, like Regnase-1, is an RNase essential for immune homeostasis but has diverged as key regulator in the IFN pathway in macrophages.

## Abstract (precis)

von Gamm et al. demonstrate that mice deficient for the RNase Regnase-3 (Zc3h12c) develop hypertrophic lymph nodes and a systemic interferon response. Regnase-3 is a functional RNase that acts in myeloid cells upon IRF signaling, suggesting it to be an evolutionary counterpart to Regnase-1.

## Graphical Abstract

**Figure:**
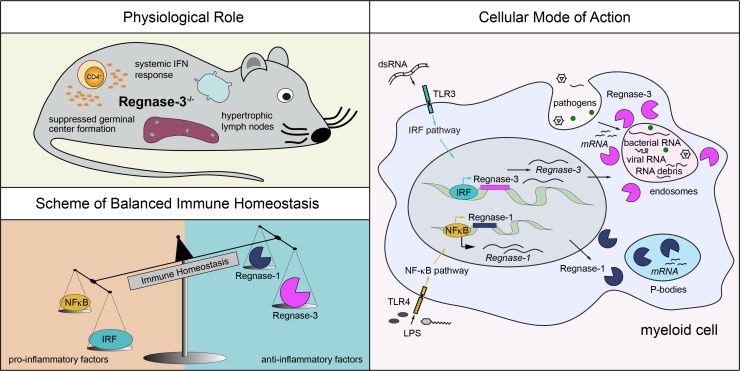

## Introduction

Immunity can be either innate or adaptive. The adaptive immune system acquires and ensures lifelong immunity against pathogens. Adaptive immune cells arise from lymphoid progenitors and comprise just three cell types: natural killer, T, and B cells. The innate immune system, on the other hand, recognizes only evolutionarily conserved pathogen-associated molecular patterns (PAMPs) and includes many cell types that arise from a single myeloid progenitor type. Innate immune cells include neutrophils, mast cells, basophils, eosinophils, dendritic cells (DCs), γδ T cells, and macrophages (Paul, 2011).

Macrophages play a major role in pathogen defense, as well as in tissue homeostasis (Okabe and Medzhitov, 2016). Macrophages engulf and digest cellular debris, microbes, and cancer cells (Hume, 2006). They have various tissue- and niche-specific functions and various provenances (Schulz et al., 2012; Amit et al., 2016; Okabe and Medzhitov, 2016). Since macrophages are among the key first-responders that recognize different PAMPs, they have to express a large variety of specific receptors. Upon activation of these receptors, a systemic intracellular immune activation occurs, enabling various defense mechanisms. Thus, macrophages initiate inflammatory responses, including the expression of certain cytokines.

Of these cytokines, IFNs are the most inflammatory and rapidly induced. IFNs are associated with intracellular infections (Faltynek and Oppenheim, 1988; Stetson and Medzhitov, 2006). The mammalian IFNs are broadly classified into type 1 (e.g., IFNα, IFNβ), type 2 (only IFNγ in human and mice), and the more recently described type 3 IFNs (also known as λ IFNs; Wack et al., 2015). IFNs are transcriptionally regulated by one of the major transcription factor families, the IFN regulatory factors (IRFs; Paun and Pitha, 2007).

The complementary major immune activation transcription factor family is NF-κB (Akira, 2013; Iwanaszko and Kimmel, 2015). NF-κB can be directly activated by various stimuli, including TLRs 1, 2, 4, 5, and 9; the cytoplasmic sensors MAF, stimulator of IFN genes (STING), and retinoic acid inducible gene I (RIG-I); and also T and B cell receptor activation. IRF activation occurs primarily in response to TLRs 3, 7, and 9 and IFN receptor activation, but also via the previously mentioned cytoplasmic sensors (Hayden and Ghosh, 2008; Akira, 2013).

Most immune signaling events activate downstream myeloid differentiation 88 (Myd88)–dependent signaling, which can lead to either NF-κB or IRF activation; thus, there is potential for crosstalk. TLR3, which recognizes double-stranded RNA (dsRNA), signals from endosomes (via the adapter TIR domain–containing adapter-inducing IFN-β [TRIF]) to the serine/threonine protein kinase 1 (TBK1), leading mainly to activation of IRF3 and IRF7, and to transcription of a selected set of genes, including type I IFNs (Akira, 2013). IRF3 can also be activated via different pathways (Liu et al., 2015), again highlighting the potential for cross talk between different signaling pathways in the immune system.

The anti-inflammatory pathways that ensure immune homeostasis are just as important as the pathways that ensure cellular and systemic responses to pathogens. Defects in the anti-inflammatory pathways that ensure immune homeostasis can lead to sterile inflammatory processes, and even to severe pathologies such as autoimmunity. The anti-inflammatory pathways that ensure immune homeostasis are just beginning to be understood. RNA-regulating factors and posttranscriptional networks are known to play a particularly important role in the interplay between pro- and anti-inflammatory pathways (Lichti et al., 2018).

One family of RNases active in immune cells, identified 10 yr ago, includes four family members that were originally named monocyte chemotactic protein-induced protein 1–4 (MCPIP1–4; Liang et al., 2008). Later studies, mostly centered on MCPIP1, revealed that the highly conserved PilT N-terminus (PIN) domain acts as an RNase; thus, MCPIP1 was renamed regulatory RNase-1 (Regnase-1; Matsushita et al., 2009; Akira, 2013). Recent studies indicate that Regnase-1 plays a broader role in the regulation of RNA beyond immune cells (Skalniak et al., 2014; Boratyn et al., 2016, 2017; Lu et al., 2016). However, phenotypic manifestations clearly show that a major, if not the primary, role of Regnase-1 is within the immune system: namely, preventing autoimmunity and ensuring pathogen defense (Iwasaki et al., 2011; Uehata et al., 2013; Mino and Takeuchi, 2015; Habacher and Ciosk, 2017). Molecular insights have revealed that Regnase-1 is an essential RNase in macrophages and in T cells, where it degrades mRNAs and viral RNAs in the cytoplasm and in foci (called P or GW bodies), which contain other mRNA regulatory factors and mRNAs. Regnase-1 is also found in the endoplasmic reticulum, where it degrades translating RNAs (Suzuki et al., 2011, Mino et al., 2015a).

Regnase-1 lacks substrate specificity in vitro*,* and it is believed that it targets specific RNAs in T cells by binding the RNA-binding protein Roquin (Jeltsch et al., 2014; Mino et al., 2015). *Regnase-1* expression is mainly regulated by NF-κB signaling: Regnase-1 is proteosomally degraded when it is phosphorylated by the IκB kinase complex (IKK) after TLR4 activation (Iwasaki et al., 2011) and is also cleaved by MALT1 upon T cell receptor activation (Uehata et al., 2013). Both major signaling events classically induce NF-κB signaling. Regnase-1 reexpression is then ensured by an integrated feedback loop wherein Regnase-1 recognizes and represses its own RNA (Iwasaki et al., 2011). Furthermore, Regnase-1 can be up-regulated by many stimuli, such as IL-17, IL-1β, and TNF signaling (Jeltsch et al., 2014; Garg et al., 2015; Mao et al., 2017; Yang et al., 2018). Overall, Regnase-1 regulation and function have evolved to regulate RNA in the NF-κB pathway in multiple ways.

The above studies have painted a complex but incomplete picture of the roles of Regnase-1, yet very little data exists for the other three Regnase proteins to either complement or extend these findings. *Regnase-4* has been knocked out in mice, which remain healthy unless challenged in a multiple sclerosis model; that study demonstrated that Regnase-4 has some role in T cell effector functions (Minagawa et al., 2014). Although in vitro overexpression data suggest that Regnase-3 might be able to regulate cell migration genes in colorectal cancer and endothelial cells (Liu et al., 2013; Suk et al., 2018), the physiological roles of Regnase-2 and Regnase-3 remain completely unknown.

A major unexplored question is whether Regnase family members are functionally redundant, or if they have evolved to acquire diverse expression or functions in immune cells. In this study, we characterized *Regnase-3* “knockout-first allele” mice and various immune cell–specific knockout mice derived therefrom. We demonstrate that, like Regnase-1, Regnase-3 is a key player in immune homeostasis but has also evolved as a key regulator within the IFN pathway in macrophages. We demonstrate that Regnase-3 can bind and degrade a variety of RNAs in vitro, but regulates only specific mRNAs (such as *Regnase-1*) in cells, although its direct targets in macrophages remain unknown. Future studies are needed to reveal the molecular mode of action of Regnase-3, which is certain to be a useful therapeutic target for the regulation of immune homeostasis.

## Results

### *Regnase-3*–deficient mice develop severe lymphadenopathy

**Figure 1.**
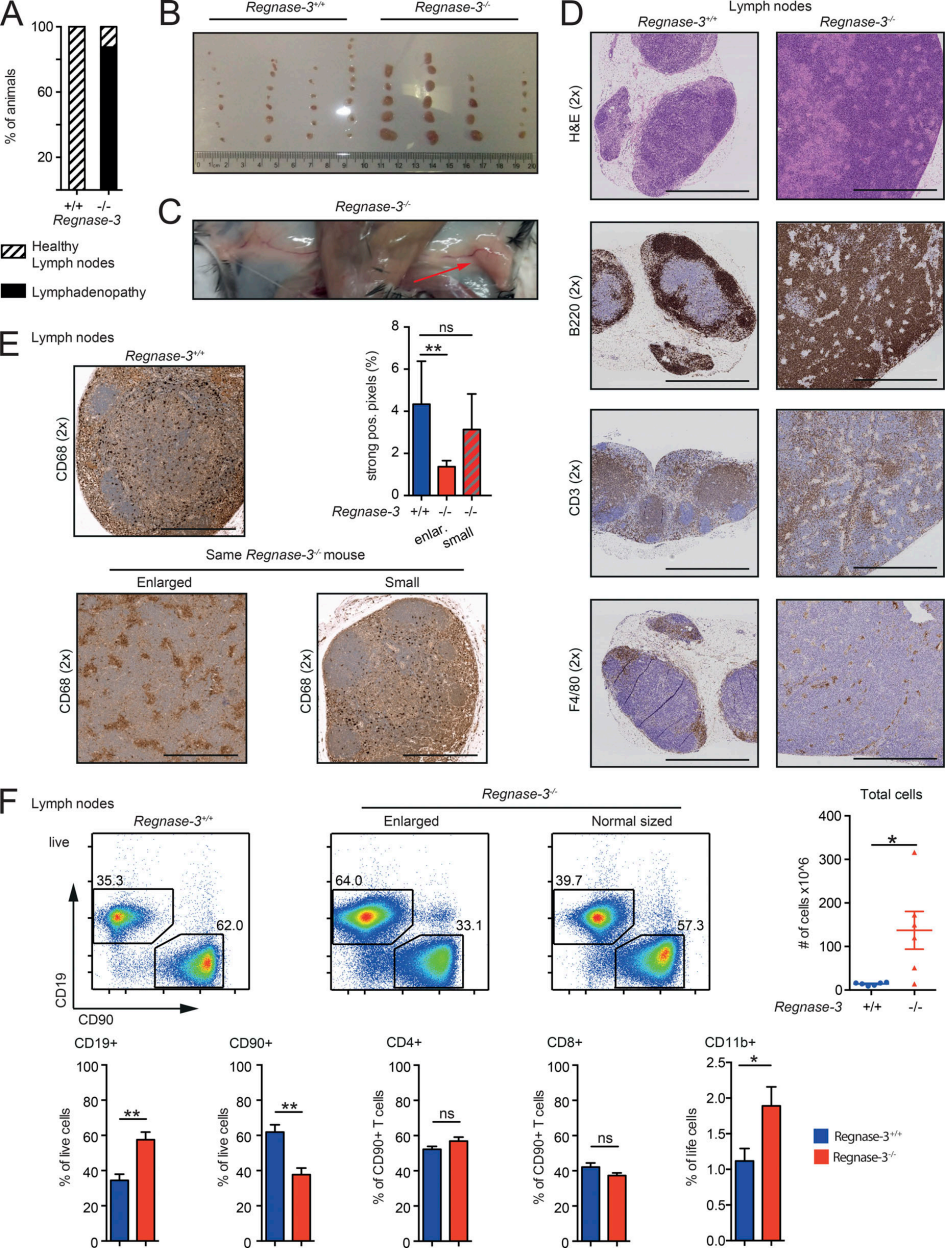
***Regnase-3*–deficient mice develop hypertrophic lymph nodes. (A)** Frequency of mice showing lymphadenopathy in a cohort of 24 *Regnase-3*^−/−^ mice and 24 *Regnase-3*^+/+^ littermate controls at 3–6.5 mo of age. **(B)** Photography of skin-draining lymph nodes of four *Regnase-3*^−/−^ mice and their *Regnase-3^+/+^* littermate controls at 5 mo of age. **(C)** Representative photography of inguinal lymph nodes of a *Regnase-3*^−/−^ mouse at 5 mo of age. Arrow indicates hypertrophic lymph node. **(D)** H&E staining and immunohistochemical analysis of B cells (B220), T cells (CD3), and macrophages (F4/80) in skin-draining lymph nodes of *Regnase-3*^−/−^ mice with lymphadenopathy and *Regnase-3^+/+^* littermate controls (representative images from *n* = 3/3). Magnification of images is indicated in brackets. Bars, 1,000 µm. **(E)** Immunohistochemical analysis of macrophages (CD68) in skin-draining lymph nodes of *Regnase-3*^−/−^ mice with lymphadenopathy and *Regnase-3^+/+^* littermate controls (representative images from *n* = 6/6). Images of enlarged and small lymph nodes are taken from the identical *Regnase-3*^−/−^ mouse. Top right: Frequency of strong positive (pos.) pixels in CD68 immunohistochemical sections of the lymph nodes was determined by Definiens software (*n* = 6/6). Bars, 500 µm. **(F)** Top: Frequencies of B cells (CD19^+^) and T cells (CD90^+^) in enlarged and normal-sized lymph nodes of the same *Regnase-3*^−/−^ mouse and its *Regnase-3*^+/+^ littermate control at 6 mo of age, assessed by flow cytometry (representative blots of *n* = 6/6). Number of total cells in lymph nodes of *Regnase-3*^+/+^ mice and *Regnase-3*^−/−^ littermates (*n* = 6/6). Bottom: Frequencies of B cells (CD19^+^), T cells (CD90^+^), CD4^+^ and CD8^+^ T cells, and CD11b^+^ cells in enlarged lymph nodes of *Regnase-3*^−/−^ mice and their *Regnase-3*^+/+^ littermate controls at 6 mo of age, assessed by flow cytometry (*n* = 6/6). Data are represented as mean ± SEM and were compared by Mann–Whitney *U* test (*, P ≤ 0.05; **, P ≤ 0.01; ns, not significant).

To study the physiological role of Regnase-3, we obtained mice with a knockout-first allele promoter-driven selection cassette, allowing both the generation of *Regnase-3*–deficient (*Regnase-3*^−/−^) mice in F2 and also conditional deletion following exposure to site-specific recombinases Cre and Flp (Fig. S1, A and B). Genotyping and RT-PCR confirmed insertion of the cassette and consequent deletion of exons 4, 5, and 6 of *Regnase-3* (premature stop; Fig. S1, C and D). Although mice were born in Mendelian ratios and had normal survival rates (Fig. S1, E and F), seven of eight *Regnase-3*^−/−^ mice developed enlarged lymph nodes (lymphadenopathy) after ∼6 mo of age (Fig. 1 A). Approximately 80% of affected *Regnase-3*^−/−^ mice exhibited lymphadenopathy of skin-draining lymph nodes (inguinal, superficial cervical, axillary, and brachial). Some mice developed only some enlarged lymph nodes mostly with a regional focus, while in some other sick mice, all lymph nodes were affected (Fig. 1, B and C; and data not shown). Enlarged lymph nodes were particularly disrupted in B and T cell zones (Fig. 1 D): CD68 staining revealed that macrophage distribution was altered and frequencies were decreased (Fig. 1 E). We performed FACS analysis to study potential shifts in major immune cell types in *Regnase-3*^−/−^ mice. We observed significantly increased frequencies of B cells (CD19^+^) in enlarged lymph nodes of *Regnase-3^−/−^* mice. The frequency of T cells (CD90^+^) was decreased; we examined both CD4^+^ and CD8^+^ cells. Due to highly increased total cell counts in the lymph nodes of *Regnase-3*^−/−^ mice, however, total cell numbers were increased for T cells (Fig. 1 F). CD11b^+^ cells, which include a variety of myeloid cells, were increased in both frequency and number in the lymph nodes of *Regnase-3*^−/−^ mice (Fig. 1 F). Total B cell numbers in pooled skin-draining lymph nodes could exceed 100 million cells in *Regnase-3*^−/−^ mice (Figs. 1 F and S1 G). In summary, *Regnase-3*–deficient mice develop severe but sublethal lymphadenopathy that is mostly characterized by an infiltration of B cells and inflammatory myeloid cells into lymph nodes.

### *Regnase-3*–deficient mice do not develop systemic autoimmunity

*Regnase-1*–deficient mice develop severe systemic autoimmunity (Akira, 2013). We therefore analyzed *Regnase-3*–deficient mice for potential signs of systemic autoimmunity.

**Figure 2.**
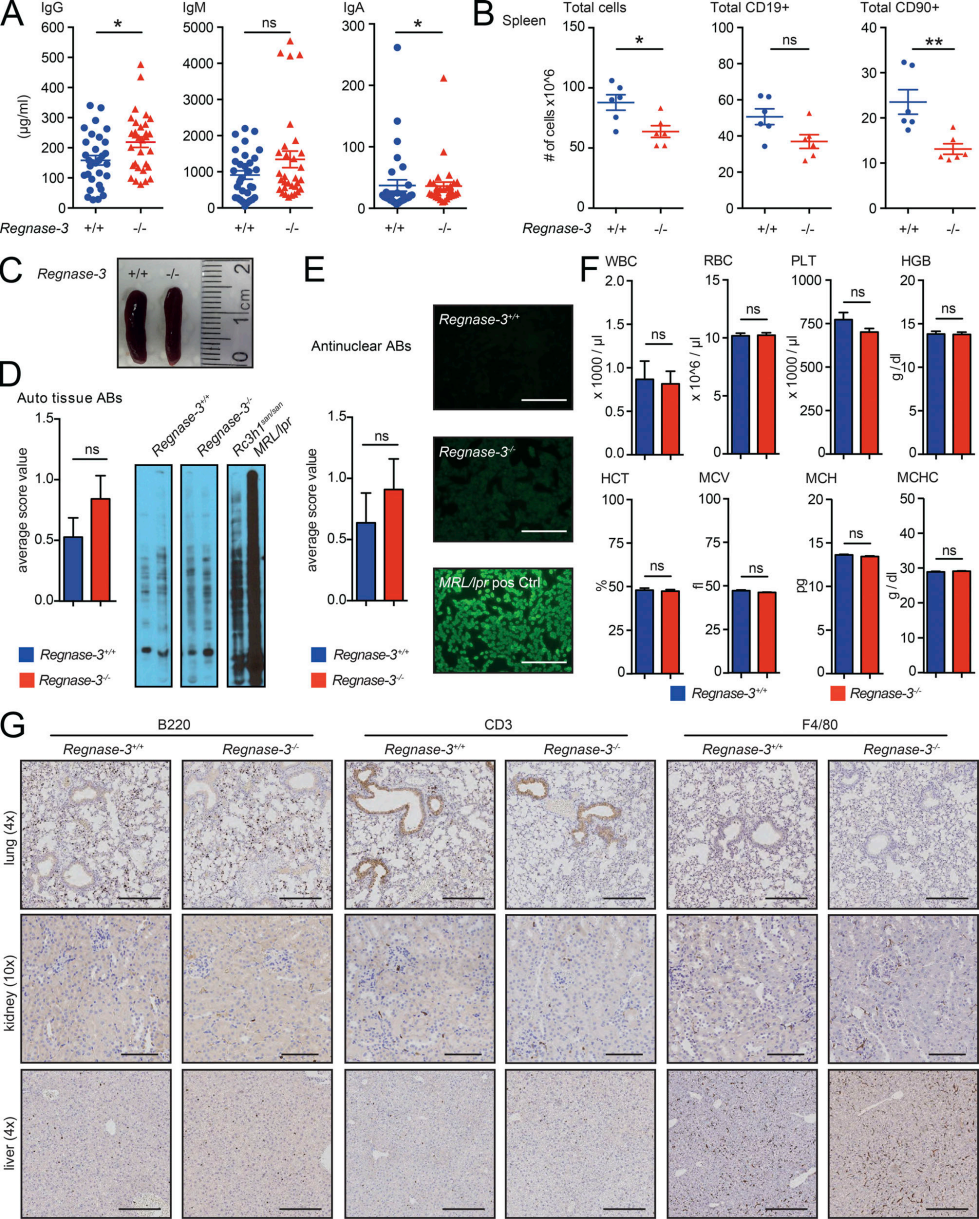
***Regnase-3*–deficient mice do not develop systemic autoimmunity. (A)** Total IgG, IgM, and IgA serum immunoglobulin levels measured by ELISA in *Regnase-3*^−/−^ mice and their *Regnase-3^+/+^* littermate controls (*n* = 31/31). **(B)** Number of total splenic cells, as well as total CD19^+^ and CD90^+^ cells, in *Regnase-3*^−/−^ mice and their *Regnase-3^+/+^* littermate controls at 6 mo of age (*n* = 6/6). **(C)** Representative photography of spleens of a *Regnase-3*^−/−^ mouse and its *Regnase-3^+/+^* littermate. *Regnase-3*^−/−^ mouse was suffering from lymphadenopathy. **(D)** Autoreactive antibodies against tissue: Liver lysates from NOD scid gamma mice were separated by SDS-PAGE and blotted to a PVDF membrane. Sera from *Regnase-3^+/+^* and *Regnase-3*^−/−^ mice were subjected to the membrane, and serum IgGs were visualized by anti-mouse IgG-HRP and scored as 0 = negative, 1 = weak positive, or 2 = strong positive (*n* = 19/19). Serum from *Rc3h1*^*san/san*^ and MRL/*lpr* mice served as positive control. Left: Statistics. Right: Representative blots. **(E)** Evaluation of antinuclear antibodies (ABs). Sera from *Regnase-3^+/+^* and *Regnase-3*^−/−^ mice were subjected to HEp-2 cells, and serum IgGs were visualized with FITC-coupled anti-mouse IgG by microscopy and scored as 0 = negative, 1 = weak positive, or 2 = strong positive (*n* = 11/11). Serum from MRL/*lpr* mice served as positive control. Left: Statistics. Right: representative images. Bar, 250 μm. **(F)** Peripheral blood counts in *Regnase-3*^−/−^ mice and their *Regnase-3^+/+^* littermate controls (*n* = 6/6). WBC, white blood cells; PLT, platelets; HGB, hemoglobin; HCT, hematocrit; MCV, mean corpuscular volume; MCH, mean corpuscular hemoglobin; MCHC, mean corpuscular hemoglobin concentration. **(G)** Immunohistochemical analysis of B cells (B220), T cells (CD3), and macrophages (F4/80) in lung, kidney, and liver sections of *Regnase-3*^−/−^ mice and *Regnase-3^+/+^* controls at 8 mo of age (representative images from three *Regnase-3*^−/−^ mice with lymphadenopathy and three *Regnase-3^+/+^* littermate controls). Magnification of images is indicated in brackets. Bars, 250 µm (lung and liver); 100 µm (kidney). Data are represented as mean ± SEM and were compared by Mann–Whitney *U* test (*, P ≤ 0.05; **, P ≤ 0.01; ns, not significant).

Serum antibody levels are massively increased in systematic autoimmunity, but we observed only a mild increase in serum immunoglobulins IgG, IgM, and IgA in *Regnase-3*^−/−^ mice (Fig. 2 A). Unlike *Regnase-1*^−/−^ mice, *Regnase-3*^−/−^ mice had normal spleens. In fact, splenocyte numbers were slightly decreased, most likely due to decreases in CD19^+^ and CD90^+^ cells (Fig. 2, B and C). To test for other forms of autoimmunity, we performed antinuclear antibody staining using tissue from immunodeficient mice for Western blot analysis and tested liver tissues for autoreactivity. Sera of *MRL/lpr* (Liu et al., 2006) and *Rc3h1^san/san^* (Vinuesa et al., 2005) mice served as controls. Neither assay indicated autoimmunity in *Regnase-3*^−/−^ mice, while both positive controls exhibited strong systemic autoimmunity (Fig. 2, D and E). Platelet, red blood cell, hematocrit, and a variety of other standard blood values were unaltered in *Regnase-3*^−/−^ mice, suggesting that their deregulated immune homeostasis is independent of autoimmunity (Fig. 2 F). Next, we tested if immune cells had infiltrated lung, kidney, or liver by staining for CD3, B220, and F4/80 (Fig. 2 G). We found no infiltration, which provides further evidence that *Regnase-3*^−/−^ mice do not develop systemic (and likely not even tissue-specific) autoimmunity.

### *Regnase-3* deficiency causes disordered primary follicles and impaired germinal center formation

**Figure 3.**
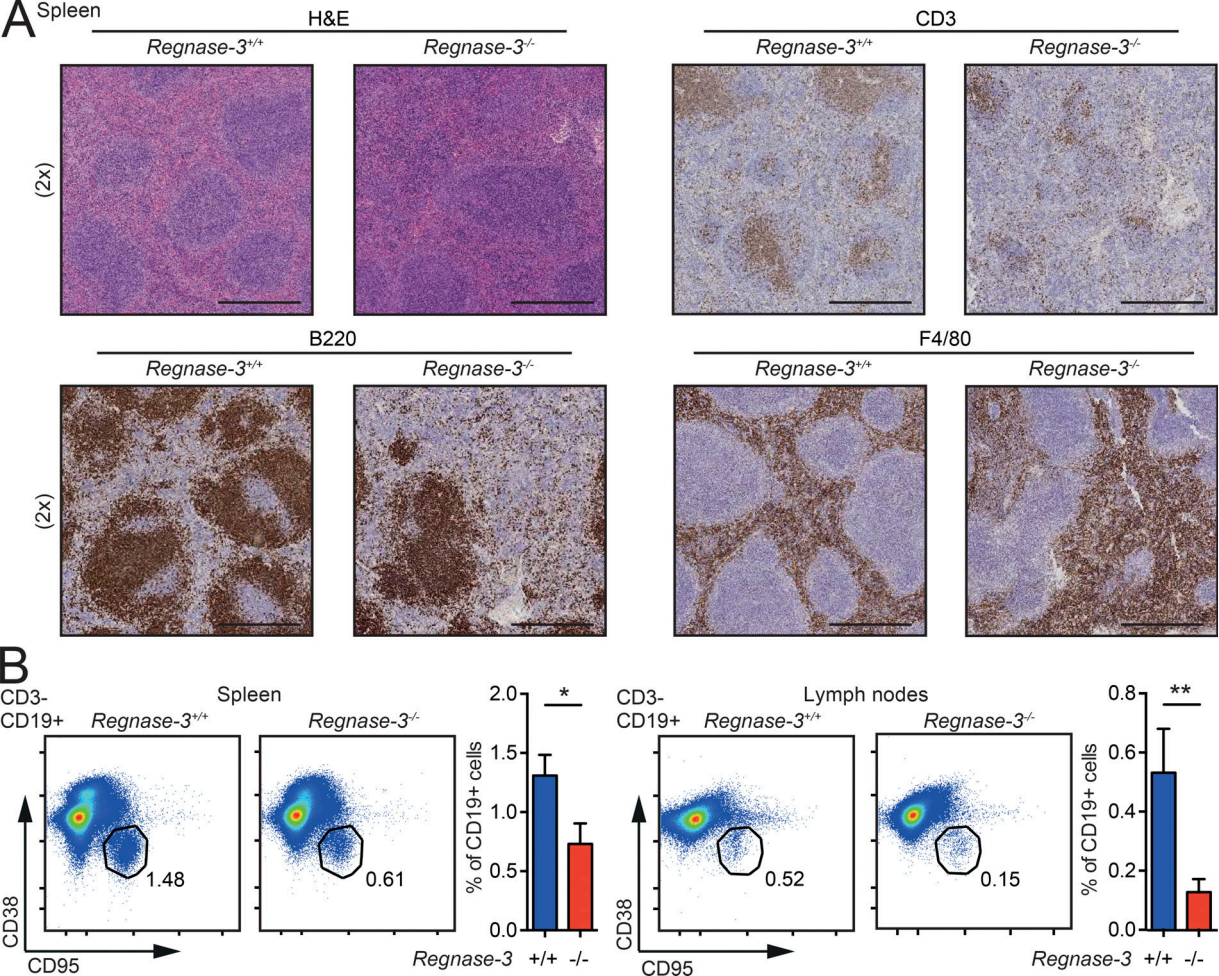
**Follicle development in Regnase-3^−/−^ mice. (A)** H&E staining and immunohistochemical analysis of T cells (CD3), B cells (B220), and macrophages (F4/80) in spleen sections of *Regnase-3*^−/−^ mice with lymphadenopathy and their *Regnase-3^+/+^* littermate controls at 8 mo of age (representative images of *n* = 3/3). Magnification of images is indicated in brackets. Bars, 500 µm. **(B)** Frequencies of germinal center B cells (CD3^−^, CD19^+^, CD38^−^/Fas^+^) in spleen and lymph nodes of *Regnase-3*^−/−^ mice with lymphadenopathy and their *Regnase-3^+/+^* littermate controls at 8 mo of age, assessed by flow cytometry (spleen, *n* = 11/11; lymph nodes, *n* = 5/5). Data are represented as mean ± SEM and were compared by Mann–Whitney *U* test (*, P ≤ 0.05; **, P ≤ 0.01).

*Regnase-1*–deficient mice develop enhanced germinal centers (Akira, 2013). Due to this and the decreased number of lymphocytes in the spleens of *Regnase-3*^−/−^ mice, we wanted to better characterize splenic microarchitecture and germinal center development. Immunohistochemical staining of the spleen for B220, CD3, and F4/80 revealed damaged follicles. Marginal zone and white pulp areas had signs of extramedullary hematopoiesis, and macrophage distribution was altered in the knockout mice (Fig. 3 A). In line with destroyed follicle development, *Regnase-3^−/−^* mice had decreased numbers of germinal center B cells in the spleen, as well as in hypertrophic lymph nodes as observed by FACS analysis (Fig. 3 B). In summary, *Regnase-3*–deficient mice do not develop systemic autoimmunity; instead, these mice display impaired follicle and germinal center formation.

### *Regnase-3* deficiency leads to increased IFN signaling

**Figure 4.**
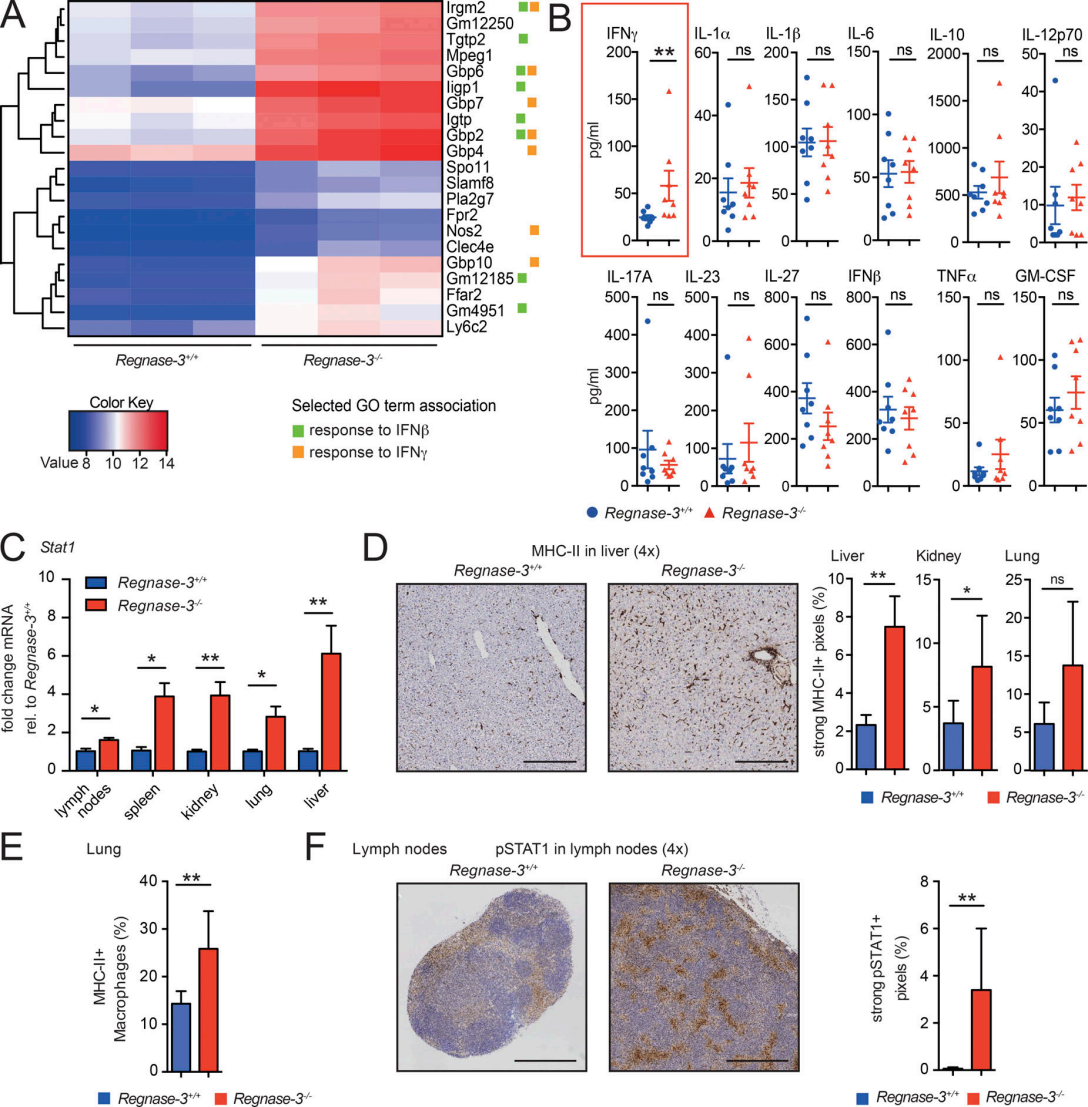
**Systemic IFN signaling in *Regnase-3*^−/−^ mice. (A)** CD19^+^ B cells were isolated from enlarged lymph nodes of *Regnase-3*^−/−^ mice and their *Regnase-3*^+/+^ littermates (*n* = 3/3), and RNA was isolated and subjected to RNA sequencing. Heatmap of RNA sequencing data for all significantly up-regulated (≥2 log2 fold) genes in *Regnase-3*^−/−^ B cells is shown. GO term association to “response to IFNβ” and “response to IFNγ” is indicated for each gene. **(B)** Serum cytokines in *Regnase-3*^−/−^ mice and *Regnase-3^+/+^* littermates at 6 mo of age (*n* = 8/8), assessed by multiplex assay. **(C)** *Stat1* mRNA expression in tissues from *Regnase-3^+/+^* and *Regnase-3*^−/−^ mice at 8 mo of age, assessed by quantitative RT-PCR, normalized to *Hprt* relative (rel.) to their expression in *Regnase-3^+/+^* mice (*n* = 5/5). **(D)** Left: Immunohistochemical analysis of MHC-II in liver sections of *Regnase-3*^−/−^ mice with lymphadenopathy and *Regnase-3*^+/+^ controls (representative images). Magnification of images is indicated in brackets. Bars, 250 µm. Right: Frequency of strong positive pixels in MHC-II immunohistochemical sections of lung, kidney, and liver were determined by Definiens software (*n* = 6 *Regnase-3*^−/−^ mice and 6 *Regnase-3*^+/+^ littermate controls). **(E)** Frequency of MHC-II–positive macrophages (CD68^+^) of all CD68^+^ macrophages in the lung of *Regnase-3*^−/−^ mice with lymphadenopathy and their *Regnase-3*^+/+^ littermate controls, assessed in immunohistochemical, consecutive sections (*n* = 5/6). **(F)** Left: Immunohistochemical analysis of pSTAT1 in skin-draining lymph nodes from *Regnase-3*^−/−^ mice with lymphadenopathy and *Regnase-3*^+/+^ littermate controls (representative images). Right: Frequency of strong positive pixels in pSTAT1 immunohistochemical sections of lymph nodes, determined by Definiens software (*n* = 6 *Regnase-3*^−/−^ mice and 6 *Regnase-3*^+/+^ littermate controls). Bars, 500 µm. Data are represented as mean ± SEM and were compared by Mann–Whitney *U* test (*, P ≤ 0.05; **, P ≤ 0.01; ns, not significant).

Although germinal center B cell development was suppressed, hypertrophic lymph nodes in *Regnase-3^−/−^* mice were marked by an infiltration of B cells. Thus, we aimed to understand the cause of imbalance in these lymph nodes. We isolated B cells from lymph nodes of three knockout mice (hypertrophic and normal lymph nodes were isolated) and littermate controls, and subjected mRNA from all samples for sequencing. Principal component analysis (PCA) demonstrated that B cells from hypertrophic lymph nodes clustered in PCA space and differentially expressed certain genes compared with other samples (Fig. S2 A). Remarkably, 12 of the 21 significantly up-regulated genes were identified as interferon (*IFNβ* and/or *IFNγ*) response genes; enrichment analysis highlighted cellular response to *IFNβ* (P < 0.05; Figs. 4 A and S2 B).

To test whether the IFN signature was systemic in *Regnase-3*^−/−^ mice, we measured a variety of classic inflammatory serum cytokines. Only IFNγ was up-regulated (Fig. 4 B). IFNs induce a variety of signaling events, including phosphorylation of STAT1, which initiates downstream transcriptional up-regulation of IFN-response genes including MHC-II genes and *Stat1* itself (in a positive feedback loop; Meissl et al., 2017). We observed significantly up-regulated *Stat1* mRNA levels in lymph nodes, spleen, kidney, lung, and liver in *Regnase-3*^−/−^ mice with lymphadenopathy (Fig. 4 C). Moreover, *Regnase-3*^−/−^ mice demonstrated increased MHC-II expression in immunohistochemistry sections from liver, kidney, and lung (which was particularly high in macrophages; Fig. 4, D and E). STAT1 phosphorylation was enhanced in hypertrophic lymph nodes of *Regnase-3*^−/−^ mice (Fig. 4 F).

In summary, the phenotypic changes observed in *Regnase-3*^−/−^ mice are consistent with both systemic and local trends toward increased IFN signaling, which can suppress follicle and germinal center formation (Heikenwalder et al., 2004). Although elevated levels of IFNγ can, under certain conditions, drive the development of autoimmunity (Jackson et al., 2016), sterile IFN signaling can suppress follicle and germinal center formation (Heikenwalder et al., 2004). This suggests that phenotypic changes in *Regnase-3*^−/−^ mice may be a consequence of elevated IFN, independent of autoimmunity.

### T and B cell abnormalities in *Regnase-3*–deficient mice are non–cell autonomous

Due to the elevated levels of IFNγ in sera, we asked whether Regnase-3 has important functions in the T cell compartment. While T cell development in the thymus (CD4^+^/CD8^+^ ratios) appeared normal (Fig. S2 C), we observed a shift within splenic T cells toward higher CD4^+^ and lower CD8^+^ T cell frequencies (Fig. S2 D). In addition, the frequencies of CD4^+^ effector T cells in lymph nodes, as well as CD8^+^ effector memory T cells in the spleen, were increased in *Regnase-3*^−/−^ mice (Figs. S2 E and 5, A and B). These results indicated an impact of *Regnase-3* deficiency on the T cell lineage.

**Figure 5.**
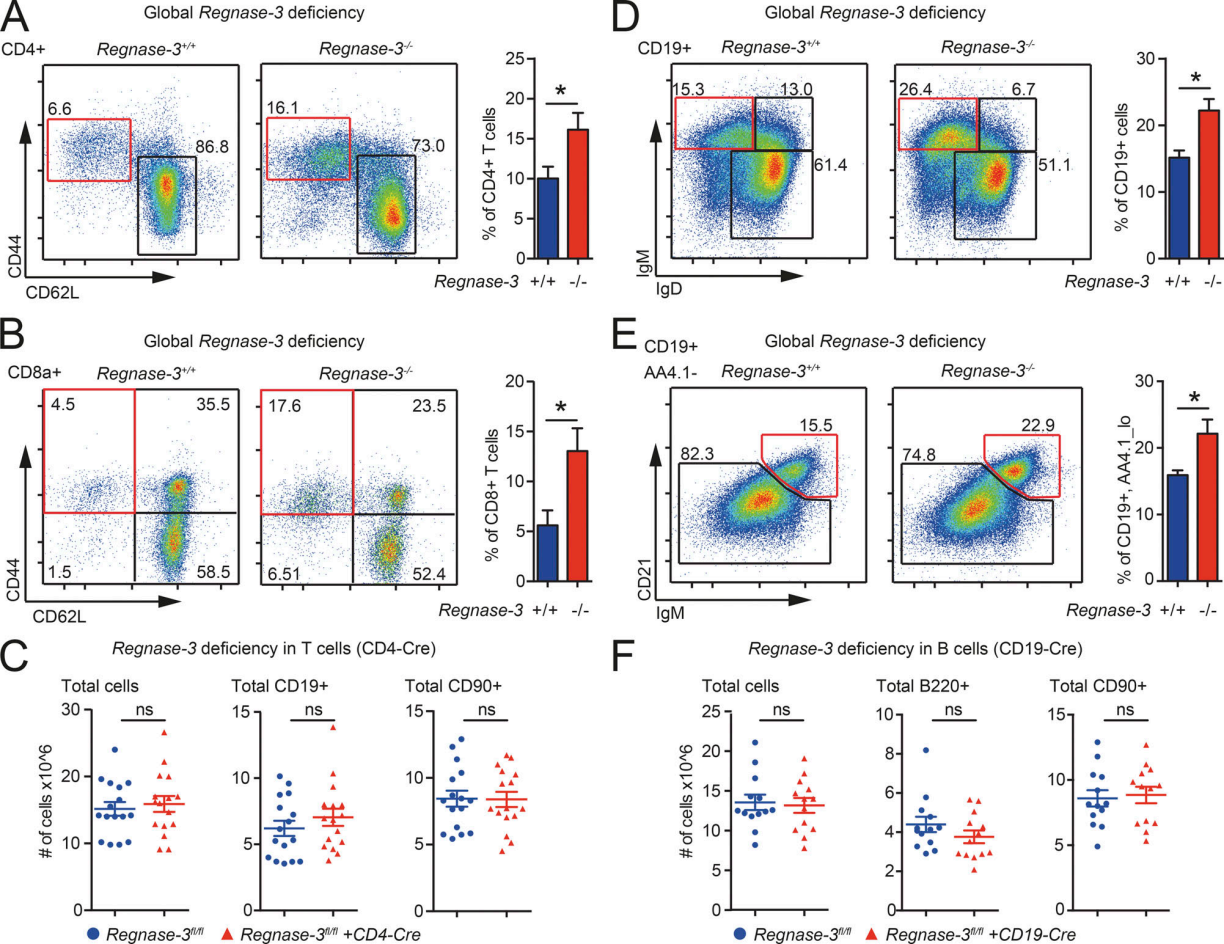
**Lymphocyte subset shifts in *Regnase-3*^−/−^ mice. (A–F)** Immune cell subsets assessed by flow cytometry. **(A)** Frequencies of CD4^+^ effector T cells (CD19^−^, CD90^+^, CD4^+^, CD62L^−^/CD44^hi^) in lymph nodes of *Regnase-3*^−/−^ mice and their *Regnase-3*^+/+^ littermate controls at 6 mo of age (*n* = 6/6). **(B)** Frequencies of splenic CD8^+^ effector memory T cells (CD19^−^, CD90^+^, CD8a^+^, CD62L^−^/CD44^hi^) of *Regnase-3*^−/−^ mice and their *Regnase-3*^+/+^ littermate controls at 6 mo of age (*n* = 6/6). **(C)** Number of total cells and calculated total B cells (CD19^+^) and T cells (CD90^+^) in lymph nodes of *Regnase-3^fl/fl^* + CD4-Cre mice and their *Regnase-3^fl/fl^* littermates at 5 mo of age (*n* = 16/16). **(D)** Frequencies of splenic immature B cells (CD19^+^, IgD^lo^/IgM^hi^) in *Regnase-3*^−/−^ mice and their *Regnase-3*^+/+^ littermate controls at 6 mo of age (*n* = 6/6). **(E)** Frequencies of splenic marginal zone B cells (CD19^+^, AA4.1^lo^, CD21^+^/IgM^hi^) in *Regnase-3*^−/−^ mice and their *Regnase-3*^+/+^ littermate controls at 6 mo of age (*n* = 6/6). **(F)** Number of total cells and calculated total B (B220^+^) and T cells (CD90^+^) in lymph nodes of *Regnase-3^fl/fl^* + CD19-Cre mice and their *Regnase-3^fl/fl^* littermates at 5 mo of age (*n* = 13/13). Data are represented as mean ± SEM and were compared by Mann–Whitney *U* test (*, P ≤ 0.05; ns, not significant).

Next, we crossed the original *Regnase-3* knockout-first allele mice with Flp recombinase expressing mice to generate *Regnase-3^fl/fl^* mice, which were used to generate conditional deletions of *Regnase-3* with cell type–specific Cre lines. To investigate if Regnase-3 executes its function intrinsically in T cells, we created conditional deletions of *Regnase-3* in CD4^+^ and CD8^+^ T cells by crossing *Regnase-3^fl/fl^* mice with CD4-Cre mice. Of note, *Regnase-1* deletion in T cells led to premature death (Uehata et al., 2013). However, specific ablation of *Regnase-3* in T cells neither affected viability nor increased lymph node size (or cell numbers; Fig. 5 C), and did not lead to any of the other severe phenotypes of *Regnase-3* deletion. Regulatory T cell numbers were unchanged in the thymus and periphery of T cell–specific *Regnase-3*–deficient mice (Fig. S2, F and G). We therefore excluded CD4^+^ or CD8^+^ cells as a causative cell type for *Regnase-3* knockout-driven lymphadenopathy.

*Regnase-3*^−/−^ mice had slightly increased frequencies of splenic marginal zone B cells and immature B cells (Fig. 5, D and E), and highly increased numbers of B cells in the lymph nodes, which appear to be primarily of a follicular phenotype (Fig. S2 H). We therefore crossed the *Regnase-3^fl/fl^* mice with CD19-Cre mice to achieve specific ablation of *Regnase-3* in B cells. These mice did not develop any obvious phenotypic abnormalities or shifts in B cell numbers, and lymph nodes were of normal size (Figs. 5 F and S2 I).

In summary, these data indicate that Regnase-3 does not exert its essential functions in T or B cells. Therefore, Regnase-3 must function in nonimmune tissues or in the myeloid lineage.

### Lymphadenopathy results from loss of *Regnase-3* in macrophages

To identify the context and cell type in which Regnase-3 acts, we analyzed mRNA levels of all *Regnase* family members in various tissues and murine immune cells upon various stimuli. Several *Regnase* family members demonstrated expression in various tissues in a mutually exclusive manner. For example, *Regnase-4* and *Regnase-1*, as expected, were specifically expressed in lymphoid organs (spleen and lymph nodes), whereas *Regnase-2* was found only in the intestine (Fig. S3 A). *Regnase-3* and *Regnase-1* mRNAs were expressed in a mutually exclusive manner in many tissues. However, these two *Regnases* were similarly regulated in various immune cells when activated with diverse inducers of TLR activation (Fig. S3 B).

**Figure 6.**
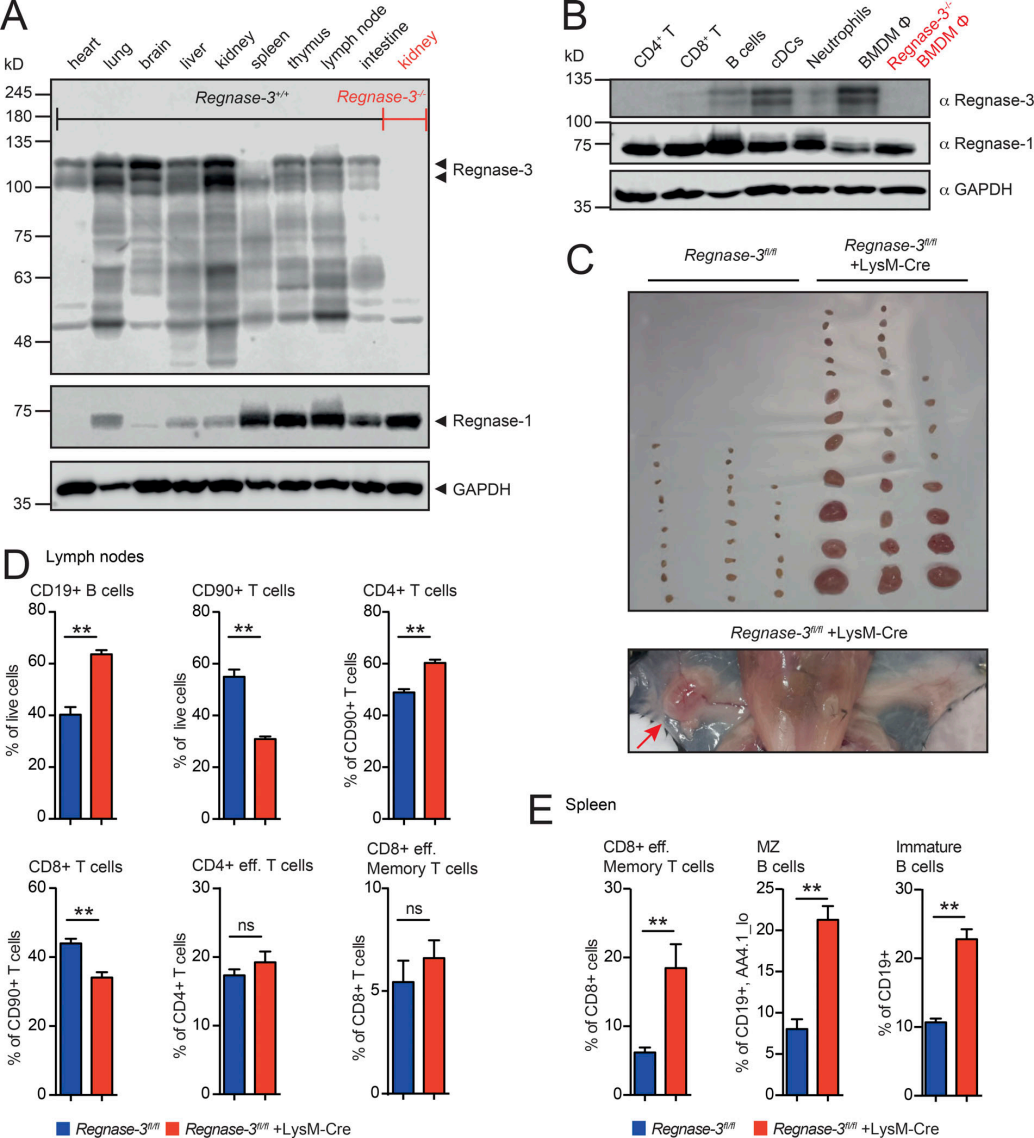
***Regnase-3* steady state expression in tissues and immune cells and phenotypic consequences on deletion in macrophages. (A)** Immunoblot analysis of Regnase-3 and Regnase-1 protein levels in indicated tissues from C57BL/6J mice (loading of 400 µg total wet tissue per lane). Kidney lysate of a *Regnase-3*^−/−^ mouse is used as a control for the specificity of the newly developed Regnase-3 antibody (representative blot of three independent experiments). Calculated molecular weights: Regnase-3, 101 kD; Regnase-1, 65 kD. **(B)** Immunoblot analysis of Regnase-3 and Regnase-1 protein levels in indicated immune cell types of C57BL/6J mice: Splenic CD4^+^ T cells, splenic CD8^+^ T cells, B cells (CD19^+^), in vitro cDCs, bone marrow neutrophils, and in vitro differentiated BMDM (Φ). Lysate of *Regnase-3*^−/−^ macrophages is shown as control (representative blot of three independent experiments). **(C)** Top: Photograph of skin-draining lymph nodes of three *Regnase-3^fl/fl^* +LysM-Cre mice and their *Regnase-3^fl/fl^* littermates at 5 mo of age. Bottom: Representative image of inguinal lymph nodes in a *Regnase-3^fl/fl^* +LysM-Cre mouse at 5 mo of age. **(D)** Frequencies of B cells (CD19^+^), T cells (CD90^+^), CD4^+^ and CD8^+^ T cells, CD4^+^ effector memory T cells (CD19^−^, CD90^+^, CD4^+^, CD62L^−^/CD44^hi^), and CD8^+^ effector memory T cells (CD19^−^, CD90^+^, CD8a^+^, CD62L^−^/CD44^hi^) in lymph nodes of *Regnase-3^fl/fl^* +LysM-Cre mice and their *Regnase-3^fl/fl^* littermate controls at 6 mo of age (*n* = 6/6). **(E)** Frequencies of splenic CD8^+^ effector memory T cells (CD19^−^, CD90^+^, CD8^+^, CD62L^−^/CD44^hi^), marginal zone (MZ) B cells (CD19^+^, AA4.1^lo^, CD21^+^/IgM^hi^), and immature B cells (CD19^+^, IgD^lo^/IgM^hi^) of *Regnase-3^fl/fl^* +LysM-Cre mice and their *Regnase-3^fl/fl^* littermate controls at 6 mo of age (*n* = 6/6). Data are represented as mean ± SEM and were compared by Mann–Whitney *U* test (**, P ≤ 0.01; ns, not significant).

To fully characterize Regnase-3 expression patterns, we generated a monoclonal antibody. This antibody strongly recognized Regnase-3 and, to a lesser extent, Regnase-2; it had no cross-reaction with Regnase-1 or Regnase-4 (Fig. S3, C and D). We considered this specificity sufficient for Regnase-3 protein analysis, since RT-PCR indicated no coexpression of *Regnase-2* and *Regnase-3* in any tissue (Fig. S3 A). Strikingly, Regnase-3 protein was highly expressed in nonlymphoid tissues, such as lung, liver, kidney, and brain. This is in clear contrast to Regnase-1, which is highly expressed in lymphoid tissues (Fig. 6 A). Notably, Regnase-3 protein migrated in at least two forms in SDS-PAGE. The lower band was in the calculated molecular weight range of 101 kD, while the upper band was 7 to 15 kD higher. Both bands were specific, as neither was seen in tissues or cells from *Regnase-3*^−/−^ mice (Fig. 6, A and B). Therefore, Regnase-3 might function within nonimmune cells, or some of the tissues expressing Regnase-3 may have a high percentage of tissue-innate immune cells, such as tissue-residing macrophages.

We probed protein from different immune cell populations with anti–Regnase-1 and anti–Regnase-3 antibodies. We found that Regnase-3 protein was, to some extent, expressed in DCs; Regnase-3 was strikingly expressed in macrophages, in which Regnase-1 is particularly sparse. As in other tissues, Regnase-3 and Regnase-1 expression levels were inversely correlated (Fig. 6 B).

To determine whether Regnase-3 acts within nonimmune tissues or, as we speculated, in macrophages, we crossed the LysM-Cre mice with *Regnase-3*^*fl/fl*^ mice. LysM-Cre achieves a broad deletion in myeloid cells, including a variety of different macrophage cell types (Abram et al., 2014), particularly tissue macrophages, as well as neutrophils, in which Regnase-3 is undetectable (Fig. 6 B). Remarkably, these mice developed the same lymphadenopathy phenotype that was observed in the mice with global *Regnase-3* deficiency (Fig. 6, C–E; and Fig. S3 E).

To further analyze the role of Regnase-3 in macrophage development, we used an in vitro system that allows tracking of the process of macrophage differentiation over time. Enforced production of Hoxb8 in this myeloid progenitor cell line can be switched off by estrogen withdrawal and, with supplemented recombinant M-CSF, Hoxb8 progenitors differentiate into macrophages (Wang et al., 2006). Strikingly, Regnase-3 was up-regulated very late upon differentiation into the macrophage lineage (Fig. S3 F). To further characterize these macrophages, we primed bone marrow–derived macrophages (BMDMs) in vitro into inflammatory (M1-like) and anti-inflammatory (M2-like) macrophages, respectively (Italiani and Boraschi, 2014) and observed slightly increased Regnase-3 expression in M1-primed macrophages (Fig. S3, G and H). Overall, our data demonstrate that Regnase-3 is specifically expressed in differentiated macrophages, where it is required to maintain immune homeostasis and avoid lymphadenopathy.

### Regnase-3 is uniquely regulated by IRF signaling

Since the phenotypic consequences of Regnase-3 deficiency suggest that Regnase-3 might play an independent role in the IFN pathway, we analyzed localization patterns in comparison to Regnase-1. We stimulated macrophages with a variety of TLR agonists and other PAMPs that were shown to regulate Regnase-1 protein expression and compared the response with Regnase-3. Regnase-1 is proteosomally degraded upon activation of IKK (Akira, 2013), which is induced when LPS activates TLR4. We therefore activated the IKK complex with LPS in the presence of the proteasome inhibitor MG132 in BMDMs, as well as in NF-κB essential modulator–deficient (NEMO) mouse embryonic fibroblasts over time. As with Regnase-1, Regnase-3 protein was found to be regulated by IKK (Fig. S4, A–C).

**Figure 7.**
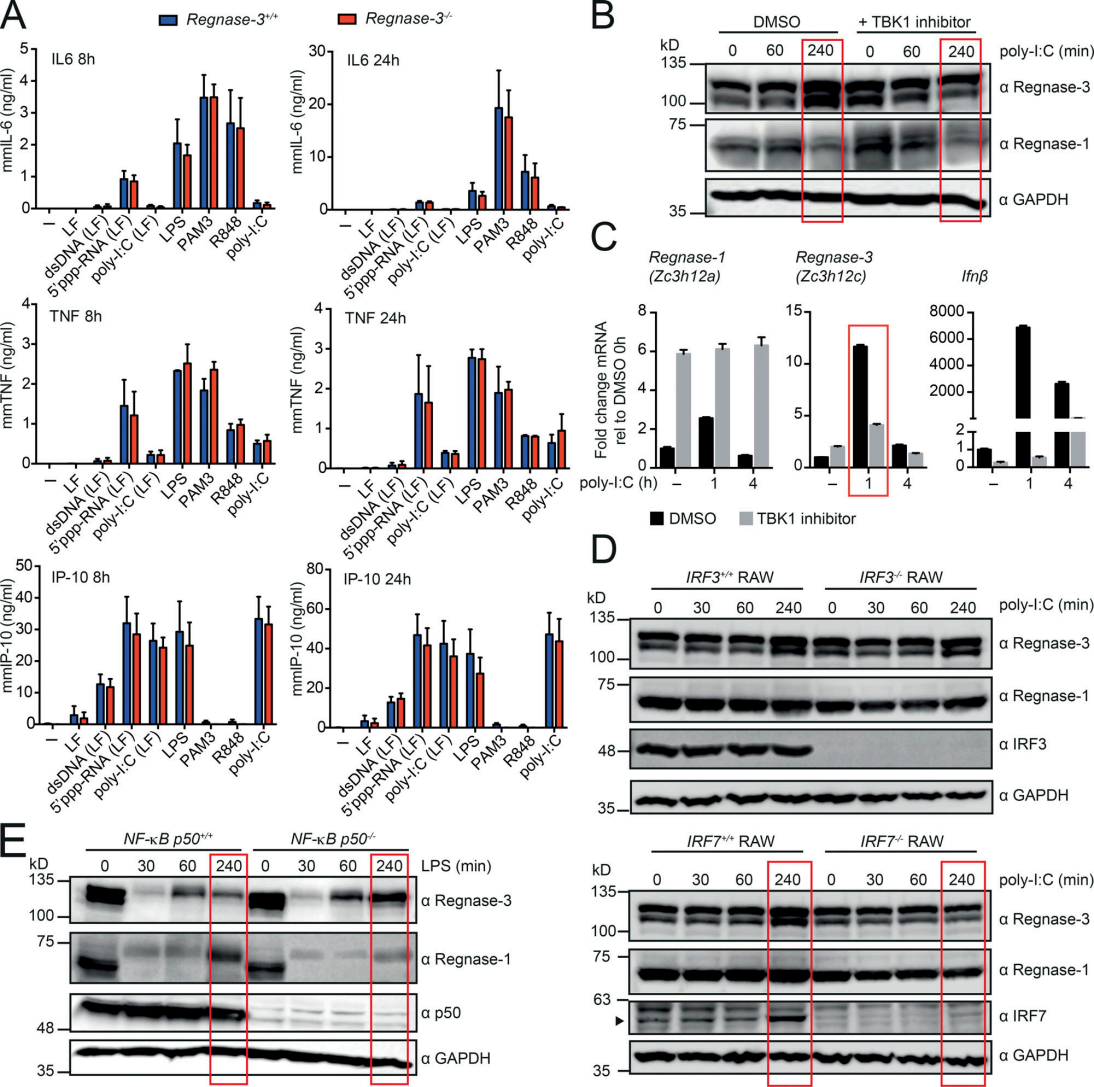
***Regnase-3* does not deregulate potential target genes and is regulated via the IFN signaling pathway. (A)** BMDMs from *Regnase-3*^−/−^ mice and *Regnase-3^+/+^* littermate controls were stimulated with dsDNA, 5′ppp-RNA, poly-I:C, LPS, Pam3CSK4 (PAM3), or R848. LF indicates that the compound was complexed to Lipofectamine 2000. 8 h and 24 h after stimulation, ELISA was used to determine IL6, TNFα, and IP-10 in the supernatant. Data are represented as mean ± SEM of three independent experiments. **(B)** BMDMs from C57BL/6J mice were preincubated for a total of 5 h with Ikkε/TBK1 inhibitor MRT67307 (20 µM) or DMSO (vehicle) and were stimulated with high molecular weight poly-I:C for indicated times and analyzed by immunoblot (representative blot of three independent experiments). **(C)** BMDMs from C57BL/6J mice were treated as in B, and *Regnase-1* (*Zc3h12a*), *Regnase-3* (*Zc3h12c*), and *IFNβ* mRNA expression was analyzed by quantitative RT-PCR, normalized to *Hprt* relative to their expression in untreated BMDMs (mean ± SEM from three biological replicates). **(D)** *IRF3*- or *IRF7*-deficient RAW cells and wild type RAW cells were stimulated with high molecular weight poly-I:C for indicated times and analyzed by immunoblot (representative blot of three independent experiments). **(E)** BMDMs from *NF-κB p50^+/+^* and *NF-κB p50*^−/−^ mice were stimulated with LPS (100 ng/ml) for indicated times and analyzed by immunoblot (representative blot from *n* = 3/3 mice).

After determining that Regnase-3 and Regnase-1 can be regulated in the same pathways, we sought to compare potential modes of action and targets. We stimulated *Regnase-3^+/+^* and *Regnase-3*^−/−^ BMDMs and quantified the secretion of the cytokines IL-6, TNFα, and IP-10. IL-6 was reported to be significantly higher secreted from *Regnase-1*–deficient macrophages, while TNFα was not (Matsushita et al., 2009), and IP-10 should indicate deregulations for the IFN secretion. We stimulated the cells with dsDNA, 5′ppp-RNA, R848, poly-I:C, LPS, or PAM3. Surprisingly, there was no deregulation of Regnase-1 targets IL-6, TNFα, or IP-10 in the *Regnase-3* knockout cells (Fig. 7 A). This result implies either that Regnase-1 can compensate for loss of Regnase-3 (we have seen Regnase-1 protein up-regulated in *Regnase-3*^−/−^ macrophages in certain contexts) or that Regnase-3 acts in a different pathway and has a different and unknown mode of action.

We next compared regulation of the two proteins via Western blot analysis using a variety of macrophage-stimulating factors, such as multiple TLR agonists and/or cytokine combinations, and found Regnase-3 protein to be regulated mainly similar to Regnase-1. The main difference in regulation was that Regnase-3 was higher expressed 4 h after poly-I:C treatment (Fig. S4, D and E). TLR3 is known to be activated by the dsRNA synthetic analogue poly-I:C; TLR3 itself activates TBK1, which activates IRF3 and IRF7 and up-regulates IFN-response genes (the type I IFNs). To confirm the regulation of Regnase-3 by TLR3, we blocked TBK1 with its specific inhibitor MRT67307. TBK1 inhibition blocked poly-I:C–mediated up-regulation of *Regnase-3* mRNA and Regnase-3 protein. In contrast, TBK1 inhibition up-regulated Regnase-1 (Fig. 7, B and C). To confirm that IRF3 and/or IRF7 transcriptionally control Regnase-3 expression, but not Regnase-1 expression, we stimulated *IRF3*^−/−^ and *IRF7*^−/−^ RAW 264.7 cells with poly-I:C. Immunoblot analysis supported our hypothesis in the case of the IRF7 deletion cells (Figs. 7 D and S4 F).

To test whether *Regnase-1* transcription, unlike *Regnase-3*, is regulated via the NF-κB pathway and not the IFN pathway, we analyzed *p50*-deficient macrophages. P50 or p52 form heterodimers with p65, which then act as functional transcription factors (Oeckinghaus and Ghosh, 2009). We activated IKK in *p50*-deficient macrophages with LPS for 4 h and followed expression of Regnase-1 and -3 by immunoblot analysis. As expected, Regnase-1 was not up-regulated in *p50* knockout cells. Regnase-3, however, was up-regulated (Fig. 7 E). Regnase-1 and Regnase-3 expression was not altered in *p50*^−/−^ macrophages upon poly-I:C treatment, which was expected, as the NF-κB pathway is not directly activated by poly-I:C (Fig. S4 G). These results demonstrate that transcription of *Regnase-1* and *Regnase-3* is regulated via different pathways, with *Regnase-1* regulated by NF-κB and *Regnase-3* by IFN signaling.

### Regnase-3 can bind 3′ untranslated regions (UTRs) and regulate gene expression

**Figure 8.**
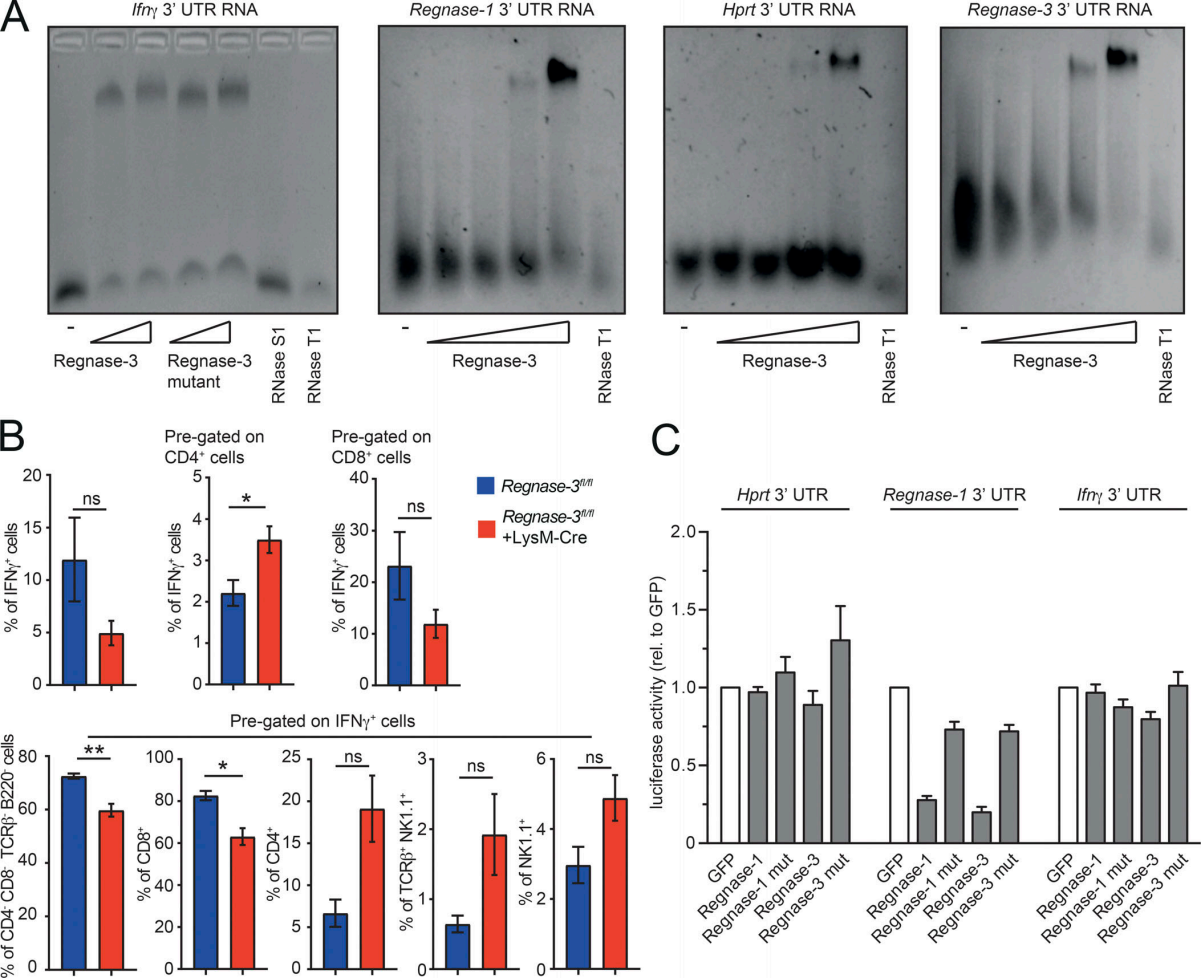
**Regnase-3 binds various 3′UTRs, regulates specific RNAs, and leads to increased IFNγ expression from CD4^+^ cells. (A)** EMSA showing Regnase-3 binding of full-length *Ifnγ* 3′UTR, *Regnase-1* 3′UTR, *Regnase-3* 3′UTR, and *Hprt* 3′UTR analyzed on a native agarose gel. Increasing concentration of Regnase-3 and its RNase inactive mutant (mut; D271N) are indicated. RNase T1 (100U) and RNase S1 (1000U) are used as positive controls. Representative gel of three independent images is shown. **(B)** Cells from lymph nodes of *Regnase-3^fl/fl^* +LysM-Cre mice with lymphadenopathy and their *Regnase-3^fl/fl^* littermate controls at 8 mo of age (*n* = 4/3) were stimulated with PMA (20 nM) and ionomycin (1 µM) for 4 h. Brefeldin A (5 µg/ml) was added for the last 2 h. IFNγ expression was analyzed by intracellular flow cytometry. Cells were stained with IFNγ, CD4, CD8, B220, TCRβ, NK1.1, and live/dead marker. Cells were pregated as indicated, and frequencies of the different subsets are displayed as mean ± SEM and were compared by unpaired two-tailed Student’s *t* test (*, P ≤ 0.05; **, P ≤ 0.01; ns, not significant). **(C)** HEK293T cells were transfected with the firefly/Renilla DuoLuc-Luciferase reporter vector of the indicated UTR together with an expression plasmid for GFP, GFP-Regnase-1, GFP-Regnase-3, or their corresponding RNase inactive mutants (D141N and D271N, respectively). Firefly luciferase activity was normalized on Renilla activity, and the regulation was calculated relative (rel.) to GFP and displayed as mean ± SEM of *n* = 3 independent experiments.

Since Regnase-1 is a well-described RNase that acts mainly as a posttranscriptional regulator (Mino et al., 2015), we examined whether Regnase-3, which shares the PIN domain and zinc finger with Regnase-1 with 97% homology, can also bind mRNA. We first analyzed *Ifnγ* and *Regnase-1* mRNA, since these proteins are deregulated in the sera of *Regnase-3*^−/−^ mice or *Regnase-3*–deficient macrophages, respectively. We also tested the *Regnase-3* 3′UTR itself, and the 3′UTR of the housekeeping gene *Hprt1* as a potential negative control. All 3′UTRs (including *Ifnγ*) were similarly bound by Regnase-3 in vitro (Fig. 8 A). Since no preferential binding was observed, and macrophages usually do not secrete IFNγ, we tried to identify the source of IFNγ, which was highlighted as the only deregulated cytokine (Fig. 4 B), in *Regnase-3^−/−^* macrophages. We therefore performed restimulation assays with spleen, small lymph nodes, and enlarged lymph nodes of *Regnase-3^fl/fl^* and *Regnase-3^fl/fl^*+LysM-Cre mice. The effects of deregulated intracellular IFNγ levels were seen only in enlarged lymph nodes (data not shown) and highlighted a higher percentage of IFNγ-producing cells within the CD4 population (Fig. 8 B). These results indicate that Regnase-3 can bind *Ifnγ* mRNA but is unlikely to regulate it. To further test this hypothesis, we performed reporter assays using transiently expressed luciferase reporter constructs with *Ifnγ*, *Hprt1*, and the *Regnase-1* 3′UTR, as well as *Regnase-3*–overexpressing constructs. We observed that the *Ifnγ* 3′UTR did not facilitate regulation by Regnase-3, while the *Regnase-1* 3′UTR was clearly down-regulated by Regnase-3, in contrast to its RNase inactive mutant (Fig. 8 C). These results align with our previous observation that Regnase-1 protein is increased in *Regnase-3*^−/−^ macrophage Western blot analysis, confirming Regnase-1 as a physiological target. In summary, although various mRNAs can be bound by Regnase-3, Regnase-3 has some specificity for certain targets under physiological conditions, which seems to be dependent on its active PIN domain.

### Regnase-3 is an RNase localized to endosomes

**Figure 9.**
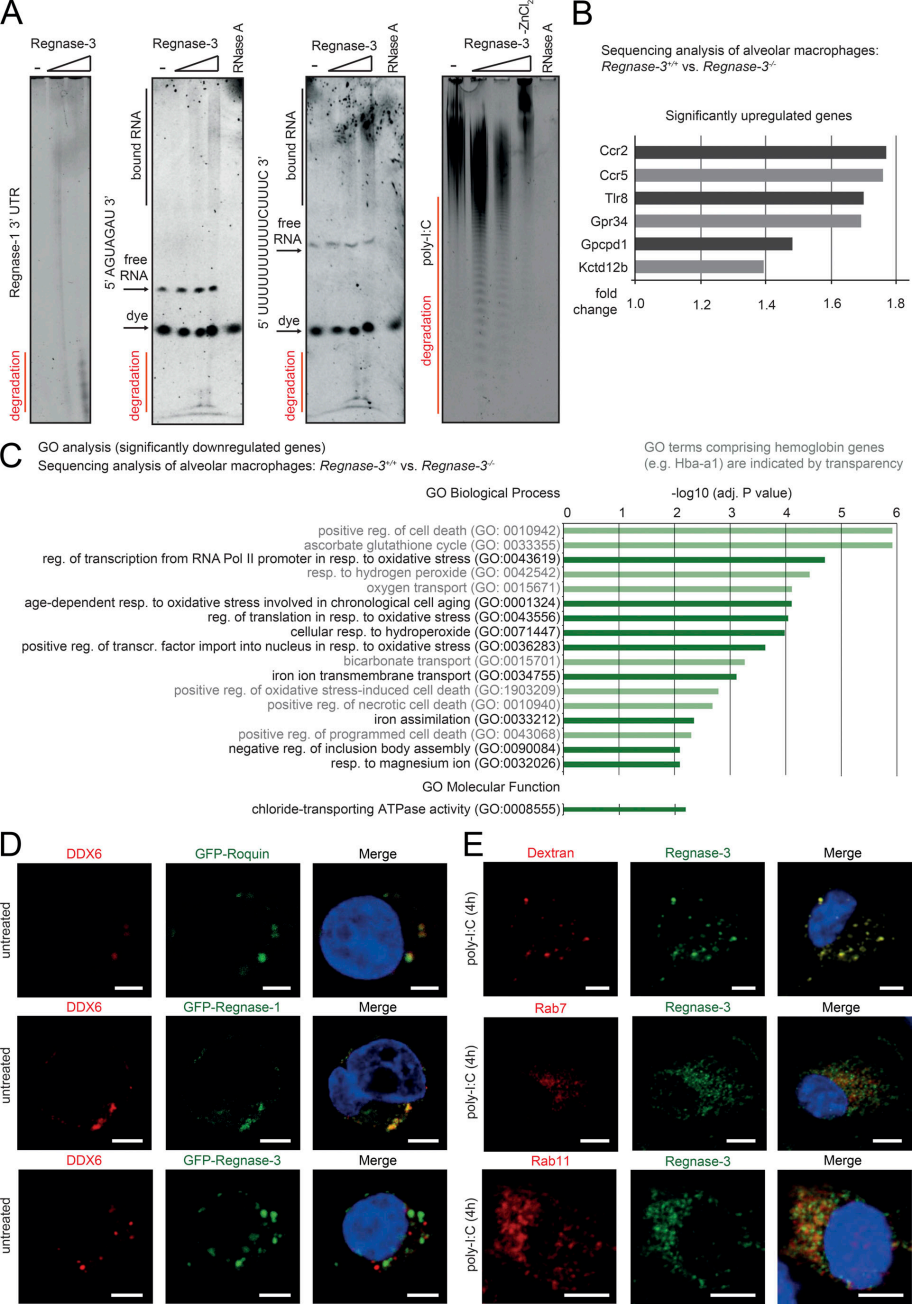
**Regnase-3 is an RNase that is localized differently from Regnase-1 in macrophages. (A)** Degradation of *Regnase-1* 3′UTR, 5′-AGUAGAU-3′, 5′-UUU​UUU​UUU​UCU​UUC-3′, and poly-I:C by Regnase-3 with ZnCl_2_ (6.93 mM) and without (–ZnCl_2_) is shown by denaturing PAGE. Increasing concentration of Regnase-3 is indicated. RNase A treatment completely degraded RNA. Degradation products, free RNA, bound RNA, and dye are indicated. **(B and C)** Alveolar macrophages (CD11c^+^) were isolated from the bronchoalveolar lavage of *Regnase-3*^−/−^ mice with lymphadenopathy and their *Regnase-3^+/+^* littermates (*n* = 3/3). RNA was isolated and subjected to RNA sequencing. **(B)** All significantly up-regulated genes (adjusted P value ≤ 0.05) in *Regnase-3*^−/−^ mice with lymphadenopathy are shown, and their respective fold-change is indicated. **(C)** GO analysis performed for all significantly down-regulated genes (adjusted P value ≤ 0.05) in *Regnase-3*^−/−^ mice with lymphadenopathy. GO terms with an adjusted P value (–log10) ≥2 are shown and listed according to it. GO terms comprising of hemoglobin genes (e.g., Hbba-a1) are indicated by transparency. Adj., adjusted; reg., regulation; resp., response; transcr., transcription. **(D and E)** Confocal microscopy images. Cells were pretreated with high molecular weight poly-I:C (10 µg/ml) for 4 h or were left untreated. Red and green as indicated; counterstain for nuclei with DAPI in blue. Representative images of three or more independent experiments are shown. Bars, 5 µm. **(D)** HEK293T cells were transfected with GFP-Regnase-1, GFP-Regnase-3, or GFP-Roquin, fixed, and immunostained for P-body marker DDX6. **(E)** Top: Dextrane Texas Red conjugate (molecular weight, 10,000 daltons) was subjected for 30 min to living BMDM cells, fixed, and stained for Regnase-3. Bottom: BMDMs were fixed and immunostained for Regnase-3 and endosomal markers Rab7 or Rab11.

We speculated that, like Regnase-1, Regnase-3 both binds and degrades RNA. Therefore, we performed gel electrophoresis under denaturing conditions to examine potentially degraded RNA products. We observed concentration-dependent degradations of the *Regnase-1* 3′UTR, short AGU repeats, UUUU repeat sequences, and poly-I:C (Fig. 9 A), indicating that Regnase-3 can degrade many different types of RNAs in vitro. Nevertheless, the actual targets of Regnase-3 in macrophages, aside from Regnase-1, remain unclear.

To obtain a better understanding of the potential mode of action of Regnase-3 in macrophages, we performed mRNA sequencing of alveolar macrophages and a detailed localization analysis of endogenous Regnase-3 in macrophages. Although some mRNAs were significantly regulated in *Regnase-3*^−/−^ alveolar macrophages, this was likely a secondary effect: the effect was slight, and most of the down-regulated genes belonged to an oxidative stress response pathway (Fig. 9, B and C).

Regnase-1 localizes to P bodies, as well as the endoplasmic reticulum, where it can regulate mRNAs (Suzuki et al., 2011; Mino et al., 2015). Our data confirmed that Regnase-1 localizes to P bodies; however, they indicated different localization for Regnase-3 (Fig. 9 D). To fully characterize the localization of Regnase-3, we used our Regnase-3 monoclonal antibody to reveal endogenous localization in macrophages in the steady state and in response to various stimuli. Regnase-3 did not localize to early endosomes characterized by EEA1 and Clathrin (at which Clathrin-mediated endocytosis takes place; Fig. S5 A). Regnase-3 only partially colocalized with the autophagosome marker LC3B (Fig. S5 B). This result indicates that Regnase-3 could localize to late endosomes or phagosomes in general, since colocalization was visible only upon TLR3 activation, which in turn leads to autophagosome fusion (Sanjuan et al., 2007). Most striking, however, was that Regnase-3 completely colocalized with Dextran Texas Red conjugates that became endocytosed, demonstrating the localization of Regnase-3 to phagosomes, and colocalized with the early endosomal marker Rab7 and Rab 11 (Fig. 9 E).

To further study Regnase-3 expression in the different stages of endocytosis, we costained late endosomes, recycling endosomes, and lysosomes. Regnase-3 partially colocalized with the late endosome and recycling endosome markers Rab7 and Rab11, respectively, but not with lysosomes (Figs. 9 E and S5, C and D). Speculating that Regnase-3 affects phagocytosis, we challenged macrophages in vitro or in vivo with *Staphylococcus aureus* but found no evidence of changes in phagocytic activity (Fig. S5, E–H). Therefore, Regnase-3 does not appear to influence phagocytic activity for bacteria per se. However, Regnase-3 can degrade multiple RNAs and localizes differently from Regnase-1, indicating its involvement in the endocytosis/phagocytosis pathway in macrophages.

## Discussion

Our data consistently demonstrate that Regnase-3 is an RNase with an important physiological role in the regulation of the IFN pathway and immune homeostasis, and that Regnase-3 is, in many ways, a functional complement to Regnase-1. This overall conclusion is supported by several facts. First, *Regnase-3* deficiency in mice causes a systemic increase in IFN signaling, with suppressed germinal center formation and no manifestations toward autoimmunity, unlike *Regnase-1* deficiency (Matsushita et al., 2009). The same phenotypic manifestations are observed when *Regnase-3* is conditionally deleted in myeloid cells. These manifestations are different from those of *Regnase-1* deletion, which results in splenomegaly, autoimmunity, and premature death, which occur upon CD4-specific deletion (Uehata et al., 2013; Li et al., 2017). Second, Regnase-3 protein is expressed in a manner complementary to Regnase-1. Regnase-3 expression is particularly high in macrophages upon TLR3 activation and is regulated by IRF3/IRF7, whereas Regnase-1 is highly expressed in lymphoid cells and is regulated via NF-κB (Iwasaki et al., 2011; Akira, 2013; Uehata et al., 2013; Uehata and Akira, 2013). Third, although Regnase-3 also binds and degrades various RNAs, unlike Regnase-1, it is localized to endosomes in macrophages, indicating a potentially unique function in the endocytic pathway.

RNA-regulating factors have various key functions within immune cells that are just beginning to be understood (Lichti et al., 2018). Depending on species, between 2% and 10% of all coding genes are believed to bind RNA (Lasko, 2000; Gerstberger et al., 2014). In the immune system, RNA-binding proteins have been particularly associated with RNA recognition and degradation. In the innate immune system, they specifically act as sensors, mostly for viruses (Pichlmair and Reis e Sousa, 2007). RNA-binding proteins are also implicated in the resolution of cellular immune responses (Lichti et al., 2018). For example, Roquin-1 and Regnase-1 control cellular immune responses and prevent autoimmunity (Matsushita et al., 2009; Glasmacher et al., 2010; Akira, 2013; Heissmeyer and Vogel, 2013). To date, most data have centered on Regnase-1 and implicated it as a key player in the regulation of RNA within the NF-κB pathway (Akira, 2013). The functional complement of Regnase-1, namely the regulator of gene expression within the IFN pathway, remained unknown.

Our data demonstrate that Regnase-3 is an RNase and the functional counterpart to Regnase-1; namely, it is a key factor in the IFN pathway in macrophages. Future work will establish the targets and thus the specific activity of Regnase-3. Our work has demonstrated that Regnase-3 has an almost sequence-independent RNase activity, similar to Regnase-1 in vitro (Matsushita et al., 2009), which is in line with its extreme high homology (97%) for the Regnase-1 PIN domain and zinc finger. Only certain mRNAs are affected by the regulation of Regnase-3 in cells. This is again in line with observations about Regnase-1, for which the functional cooperative partner was suggested to be Roquin-1 to ensure specificity (Matsushita et al., 2009). Although we confirmed that Regnase-1 was a real target for Regnase-3 in macrophages, which is regulated in reporter assays as well as endogenously in macrophages, it is unlikely that Regnase-1 is the only target that would explain the specific phenotypic signature resulting from *Regnase-3* deletion. It therefore remains unclear which RNAs are targeted by Regnase-3 and whether Regnase-3 degrades intracellular mRNAs or incoming RNA from cell debris or viruses. Based on the observation that *Regnase-3*–deficient mice exhibit malfunctions in IFN signaling, which is one of the major signatures of viral infection (Stetson and Medzhitov, 2006), the latter may be the major target of Regnase-3.

The function of Regnase-3 in macrophages and immune regulation also remains to be conclusively determined. Based on certain molecular observations, Regnase-3 could be involved in degrading extracellular material. First, the localization of Regnase-3 to late endosomes suggests that phagocytized RNA could be degraded by Regnase-3. Second, *Regnase-3*–deficient macrophages demonstrated only slightly deregulated gene expression upon various challenges, including TLR3 agonist poly-I:C (sequencing data not shown). Although macrophages isolated from lung tissues exhibited alterations in gene expression, these indicated cellular changes due to hypoxic stress and, potentially, changes secondary to an infection. It is possible that Regnase-1 compensates for Regnase-3 deficiency under certain conditions, given that Regnase-1 plays a role in macrophages: LysM-Cre–specific deletion of *Regnase-1* leads to splenomegaly, increased cytokine secretion, and lung injury upon LPS challenge (Li et al., 2017).

However, the fact that no mRNAs were affected by *Regnase-3* deficiency in macrophages could also occur if Regnase-3 simply was not activated (or remained in an inactive state) in BMDMs stimulated with TLR and other PAMP agonists. A lack of activation is suggested by observations for certain other RNases of immune cells. For example, RNase L is active only when 2′-5′ oligoadenylate synthetase is activated (Pichlmair and Reis e Sousa, 2007); it could be that *Regnase-3* needs such a specific activation pattern, and that this activation pattern remains to be identified.

Another possible explanation for the lack of effect on differential gene expression in BMDMs stimulated with various agonists is that Regnase-3 recognizes only specific structures in pathogenic RNA. RNA-binding specificity has been demonstrated for many sensing molecules, including the TLRs and RIG-I (Pichlmair and Reis e Sousa, 2007); this agrees with the hypothesis of extracellular/phagocytosed RNAs as Regnase-3 targets. However, many RNases are relatively unspecific, and often achieve specificity via their regulation and interaction with other proteins, as has been suggested for Regnase-1 (Matsushita et al., 2009). In line with this explanation, we observed that Regnase-3 can degrade multiple RNAs, at least in vitro. Whether there is a preferential activity pattern remains to be determined. The C-terminal end of Regnase-3 is the only part of the protein that is not homologous to Regnase-1. It is possible that the Regnase-3 C-terminus facilitates unique protein interactions that may localize Regnase-3 to endosomes, thereby enabling a unique mode of action. It should also be mentioned that it remains to be determined whether Regnase-3 has some function within nonimmune tissues, as Regnase-3 is extremely highly expressed in some of these tissues.

Overall, our results demonstrate that Regnase-3 is a key regulatory factor in the IFN pathway in tissue macrophages and is important to maintain cellular and systemic homeostasis. Indeed, polymorphisms in *Regnase-3* have been associated with psoriasis in genome-wide association studies (Tsoi et al., 2012). Our results indicate that Regnase-3 may be an important potential therapeutic target for diseases associated with tissue inflammation and deregulation of the IFN pathway, and therefore warrant future studies on the molecular mode of action of Regnase-3.

## Materials and methods

### Mice

Mice with knockout-first allele for *Regnase-3* (Zc3h12c^tm2a(EUCOMM)Hmgu^ mice) were designed and generated by EUCOMM on C57BL/6 background through targeted gene disruption using electroporation of the linearized L1L2_Bact_P vector, harboring the promoter-driven cassette together with 5′ and 3′ arms of the *Zc3h12c* locus (Fig. S1 A). The introduced cassette harbors a splice acceptor and a premature poly-A signal between exons 3 and 4 and therefore produces a destructed *Zc3h12c* gene product (mice are termed *Regnase-3*^−/−^ herein; Fig. S1, A and D). PCR analysis of mouse ear tissue was used to identify the genotype, using wild type primers (*Zc3h12c*_WT_fw, 5′-CTG​GCT​GAC​AGA​AAT​ATC​TGT​C-3′; *Zc3h12c*_WT_rev, 5′-GGT​GCT​CAG​ACT​TCA​ACC​TG-3′) and primers to identify the cassette (*Zc3h12c*_KO_fw, 5′-GGA​AGA​AGT​TCA​TAG​ATG​AGC​GG-3′; *Zc3h12c*_KO_rev, 5′-GAA​CTG​ATG​GCG​AGC​TCA​GAC-3′; Fig. S2 C). To generate mice with floxed alleles for *Regnase-3* (*Regnase-3^fl/fl^*), ROSA26-Flp deleter mice (*Gt(ROSA)26Sor*^tm1(FLP1)Dym^) were crossed to Zc3h12c^tm2a(EUCOMM)Hmgu^ mice, and the Flp transgene was eliminated in the following generations (Fig. S1 B). *Regnase-3^fl/fl^* mice were crossed to CD4-Cre mice (STOCK Tg(Cd4-cre)1Cwi/BfluJ) to generate *Regnase-3* ablation in T cells (*Regnase-3^fl/fl^* +CD4-Cre). *Regnase-3^fl/fl^* mice were crossed to CD19-Cre mice (C.Cg-*Cd19^tm1(cre)Cgn^ Igh^b^*/J) to generate *Regnase-3* ablation in B cells (*Regnase-3^fl/fl^* +CD19-Cre). *Regnase-3^fl/fl^* mice were crossed to LysM-Cre mice (B6.129P2-*Lyz2^tm1(cre)Ifo^*/J) to generate *Regnase-3* ablation in the myeloid linage (*Regnase-3^fl/fl^* +LysM-Cre). *Regnase-3^fl/fl^* mice were crossed to ROSA26-Cre mice (GT (ROSA) 26Sor) to generate global deletion of exon 4, and the Cre transgene was eliminated in the following generations. These mice were used in some assays alternatively to mice with knockout-first allele, in particular in in vitro experiments, as both mice are globally deficient for Regnase-3. p50^−/−^ mice (B6.Cg-*Nfkb1^tm1Bal^*/J) were described elsewhere (Sha et al., 1995). C57BL/6J mice (from The Jackson Laboratory) and all other mentioned mouse lines were bred at a specific pathogen–free barrier animal facility of the Helmholtz Center Munich in accordance with institutional as well as state and federal guidelines. Animal experimental protocols were approved by the regulations of the governmental district of Upper Bavaria (protocol numbers ROB-55.2-2532.Vet_02-14-33, 55.2–1-54-2532-190-2015, and AZ 55.2–1-54-2532-215-2014).

### Cloning and vectors

Coding sequences for *Regnase-1-4* (*Zc3h12a-d*) were obtained from GeneArt (Life Technologies) or IMAGE clones (Source BioScience). Coding sequences were cloned into pCR8/GW/TOPO (Thermo Fisher Scientific), and the eGFP sequence (obtained from GeneArt) was inserted with restriction sites to obtain an N-terminal fusion protein. RNase inactive mutants were generated with the QuikChange II site directed mutagenesis kit (Agilent Technologies). For mammalian expression, *GFP-Regnase* constructs were subcloned into pLNCX2 and pMSCV/Puro. Similarly, *Regnase-3* constructs with N-terminal Flag-tag were generated. For bacterial expression, nucleotides corresponding (aa 1–478) in the Regnase-3 protein sequence were cloned as a fusion protein with N-terminal glutathione S-transferase (GST)–tag and C-terminal His-tag into pGEX-6P-2. Sanger sequencing verified integrity of the cloned coding sequences. *GFP-Roquin-1* in pDEST12.2 was kindly provided by Vigo Heissmeyer (Ludwig-Maximilians-Universität München, Munich, Germany). For luciferase measurements, the 3′UTRs of hypoxanthine-guanine phosphoribosyltransferase (*Hprt*; NM_013556.2), *Ifng* (NM_008337.4), or *Zc3h12a* (NM_153159.2) mRNAs were inserted downstream of the stop codon of the firefly luciferase in the firefly/Renilla Duo-Luciferase reporter vector (pEZX-MT06; GeneCopoeia).

### Cell culture

HEK293T cells were obtained from the American Type Culture Collection, L929 cells were kindly provided by Prof. Henriette Uhlenhaut (Helmholtz Center Munich, Munich, Germany), stem cell factor–producing Chinese hamster ovary cells were provided by Christian Schulz (Ludwig-Maximilians-Universität München, Munich, Germany) RAW-Lucia ISG cells, RAW-Lucia ISG-KO-IRF3 cells, and RAW-Lucia ISG-KO-IRF7 cells were obtained from InvivoGen. MEF cells and estrogen receptor–driven Hoxb8-expressing bone marrow progenitor (ER-Hoxb8) cells are described below. HEK293T cells were maintained in DMEM (Life Technologies), supplemented with 10% FBS (Life Technologies), 10 mM Hepes, pH 7.2 (Life Technologies), and 100 U/ml penicillin/streptomycin (P/S; Life Technologies). Jurkat cells were maintained in Roswell Park Memorial Institute (RPMI) 1640 medium (Life Technologies), supplemented with 10% FBS, 10 mM Hepes, pH 7.2, and 100 U/ml P/S. L929 cells were maintained in DMEM (Sigma-Aldrich), supplemented with 10% heat-inactivated FBS (Sigma-Aldrich) and 100 U/ml P/S. RAW cells were maintained in DMEM, supplemented with 10% heat-inactivated FBS, 100 U/ml P/S, and 10 mM Hepes, pH 7.2. All cells were kept at 37°C, 5% CO_2_.

### ER-Hoxb8 cells

ER-Hoxb8 cells were generated as described previously (Redecke et al., 2013). The pMSCV retroviral vector expressing an ER-Hoxb8 was kindly provided by Hans Haecker (Department of Infectious Diseases, St. Jude Children’s Research Hospital, Memphis, TN). To differentiate macrophages, ER-Hoxb8 progenitor cells were harvested, washed twice in PBS to remove residual β–estradiol, and resuspended in RPMI 1640, supplemented with 10% FBS, 30 µM 2-mercaptoethanol, 100 U/ml P/S, 1% cell culture supernatant from a stem cell factor–producing Chinese hamster ovary cell line, and 20 ng/ml M-CSF (Peprotech). Cells were differentiated for 0 to 8 d on non–tissue culture–treated 8-cm dishes, and cells of each plate were counted, lysed in 2× concentrated boiling Laemmli buffer, and analyzed as described in Immunoblotting.

### MEFs

MEFs deficient for *NEMO* (*NEMO^−/−^* MEF) and *NEMO*-reconstituted MEFs (*NEMO^+/+^* MEF) were kindly provided by Daniel Krappmann (Helmholtz Center Munich, Neuherberg, Germany). To obtain cell lines with constitutive overexpression of GFP-tagged Regnase family members, *NEMO^+/+^* and *NEMO^−/−^* MEFs were transduced with retrovirus as follows. To generate the retrovirus, HEK293T cells were transfected by the calcium phosphate method with plasmids containing Regnase family members with GFP tag in pMSCV/Puro backbone (see Cloning and vectors) together with the retroviral packaging plasmid EcoPack. 2 d later, the supernatant was sterile filtered using 0.45-µm syringe filters (Millipore) and supplemented with 10 µg/ml Polybrene (hexadimethrine bromide; Sigma-Aldrich). Viral supernatant was applied to MEF cells for 8 h, and the transduction procedure was repeated several times. Positive cells were selected using 2 µg/ml Puromycin antibiotics (Merck). MEF cells were kept in MEF maintenance medium (DMEM [Life Technologies] supplemented with 10% FBS, 100 U/ml P/S, and 10 mM Hepes, pH 7.2). *NEMO^+/+^* and *NEMO^−/−^* MEFs were stimulated with 1 µg/ml LPS from *Salmonella enterica* serotype typhimurium (Sigma-Aldrich) for indicated times and analyzed by immunoblot (see Immunoblotting).

### Flow cytometry

Lymphoid organs were cut into pieces and strained through a 70-µm mesh (Falcon). Erythrocytes were lysed in Tris–ammonium chloride buffer (2.06 g/liter Tris and 7.47 g/liter NH_4_Cl, pH 7.2), and cells were resuspended in surface-staining buffer (PBS containing 0.5% BSA, 0.01% sodium azide, and 2 mM EDTA). Fc receptors were blocked by preincubation with anti-CD16/32 (FCR3-4G8; in-house). Cells were first stained with the fixable viability dye Zombie Aqua (BioLegend) or eFluor 780 (eBioscience) and then surface-stained with antibodies from BioLegend: anti-CD3e (145-2C11), anti-CD4 (GK1.5), anti-CD8a (53-6.7), anti-CD11b (M1/70), anti-CD11c (N418), anti-CD19 (6D5), anti-CD23 (B3B4), anti-CD38 (8D9), anti-CD45R/B220 (RA3-6B2), anti-CD62L (MEL-14), anti-CD90.2/Thy1.2 (53–2.1), anti-CD95 (15A7), anti-CD206 (MMR), anti-F4/80 (BM8), anti-Gr1 (RB6-8C5), anti-IgD (11-26c.2a), and anti-IgM (RMM-1); antibodies from BD Biosciences: anti-CD4 (RM4-5); antibodies from BD PharMingen: anti-CD44 (IM7); and antibodies from eBioscience: anti-NK1.1 (PK136), anti-CD21 (8D9), anti-CD93/AA4.1 (AA4.1), anti-TCRβ (H57-597), and anti-B220 (RA3-6B2). Cells were then fixed in 4% paraformaldehyde (PFA) in PBS. For intracellular staining of FoxP3, cells were first surface stained, then fixed with a FoxP3 staining buffer set (eBioscience) and stained with anti-FoxP3 (FJK-16s; eBioscience) according to the manufacturer’s protocol. For intracellular IFNγ cytokine staining, cells were stimulated with PMA (20 nM) and ionomycin (1 µM) for 4 h, and brefeldin A (5 µg/ml) was added for the last 2 h. Cells were stained with fixable viability dye for 20 min at 4°C followed by surface marker staining with the appropriate antibodies in surface staining buffer for 30 min at 4°C. Cells were fixed in 4% PFA in PBS for 8 min at room temperature and permeabilized in 0.5% saponin buffer containing 1% BSA. Intracellular staining antibody anti-IFNγ (XMG1.2) was added, incubated for 30 min at 4°C, and washed with intracellular staining buffer. Cells were kept in surface staining buffer until measured by flow cytometry. Samples were analyzed on a FACS AriaIII flow cytometer (BD Biosciences), and data were analyzed with FlowJo software (versions 9 and 10).

### Immunohistochemistry

2-µm sections of 4% PFA-fixed (Roti-Histo-Fix 4%) and paraffin-embedded murine tissues were processed for immunohistochemistry. Antigen retrieval was performed with EDTA solution for B220, CD3, ki67, and phospho-STAT1 antigens and with proteolytic solution (AR9551; Leica) for MHC-II and F4/80 antigens. Immunostaining was performed on the immunohistochemistry robot Bond Max (Leica) using the antibodies stated below and the Bond Polymer Refine Detection Kit (including peroxidase block, secondary HRP antibody, 3,3′ diaminobenzidine reagent, and H&E; DS9800). Sections were dehydrated using the following steps: H_2_O, 70% ethanol, 100% ethanol (2×), and xylol (2×) and mounting medium was applied. Staining antibodies were anti-B220 (RA3-6B2, rat-IgG2a; BD), anti-CD3 (SP7, rabbit IgG; Zytomed), anti-F4/80 (BM8, rat-IgG2a; Linaris), anti-MHC-II (M5/114.15.2, rat IgG; Novus Biologicals), anti-CD68 (Ab125212, rabbit IgG; Abcam) anti-ki67 (SP6, rabbit IgG; Thermo Fisher Scientific), and anti-Tyr701-phospho-STAT1 (58D6, rabbit IgG; Cell Signaling). If primary antibody was from rabbit, secondary anti-rabbit HRP antibody from Bond Max kit was used. If primary antibody was from rat, an additional bridging antibody, rabbit anti-rat (312-005-045; Dianova), was used before anti-rabbit HRP incubation. Whole digital slides were acquired using the SCN400 slide scanner (Leica) or the AxioScan.Z1 digital slide scanner (Zeiss). Images were evaluated using the commercially available image analysis software Tissue Studio version 4.4 (Definiens) following a previously published procedure (Feuchtinger et al., 2015).

The frequencies of MHC-II^+^ macrophages were determined by counting all macrophages and MHC-II^+^ macrophages in consecutive immunohistochemical sections of ∼73,000-µm^2^ area of a representative lung in each analyzed animal.

### RT-PCR

Samples were lysed with Trizol lysis reagent (Qiagen), and whole tissues were additionally processed with the TissueLyser bead mill (Qiagen). Total RNA was extracted according to the manufacturer’s instructions. cDNA was synthesized using QuantiTect reverse transcription kit (Qiagen) according to the manufacturer’s instructions. If not stated otherwise, power SYBRgreen Master mix (Thermo Fisher Scientific) was used to perform the real-time PCR on a QuantStudio 6 real-time PCR system (Thermo Fisher Scientific). Threshold cycle values were normalized to *Hprt* and amplification efficiency for *Hprt*, *Zc3h12a*, *Zc3h12b, Zc3h12c*, and *Zc3h12d* was included. The RT-PCR amplification efficiency for *Hprt*, *Zc3h12a*, *Zc3h12b, Zc3h12c*, and *Zc3h12d* was calculated based on the slope of the standard curve and was within the range of 90–100%. The following primers (forward; reverse) were used: *Hprt* (5′-AGC​AGT​ACA​GCC​CCA​AAA​TG-3′; 5′-ATC​CAA​CAA​AGT​CTG​GCC​TGT-3′), *Zc3h12a* (5′-GTC​ATC​GAC​GGA​AGC​AAT​GT-3′; 5′-ATA​TCT​GTG​TGG​CCT​CGC​TC-3′), *Zc3h12b* (5′-TGC​TGA​GCT​GGA​CAG​AGA​GA-3′; 5′-CAG​TCC​ATC​ATG​GCA​GTG​AAT-3′), *Zc3h12c* (5′-GAA​CAG​TCC​CGC​CCT​GAC-3′; 5′-CAT​CAT​AGC​ACA​CCA​CTC​GC-3′), *Zc3h12d* (5′-GAG​CCA​TCA​AGG​TCT​GAT​ACT​CC-3′; 5′-TCA​TCG​TAG​CAG​ACC​ACT​CG-3′), *Ifnb* (5′-TGG​GAG​ATG​TCC​TCA​ACT​GC-3′; 5′-CCA​GGC​GTA​GCT​GTT​GTA​CT-3′), and *Stat1* (5′-ACA​ACA​TGC​TGG​TGA​CAG​AGC​C-3′; 5′-TGA​AAA​CTG​CCA​ACT​CAA​CAC​CTC-3′).

### Luciferase reporter assay

HEK293T cells were transfected with the firefly/Renilla DuoLuc-Luciferase reporter vector and an expression plasmid for *GFP*, *GFP-Regnase-1*, or *GFP-Regnase-3* as indicated via the calcium phosphate method. 24 h later, the cells were lysed, and firefly and Renilla activity was determined according to the manufacturer’s protocol of the LucPair Duo-Luciferase Assay Kit 2.0 (GeneCopoeia). Firefly activity was normalized to the Renilla activity.

### Immunoblotting

Whole-tissue lysates were generated by snap-freezing organs in liquid nitrogen and grinding in a mortar. Tissue powder was weighed and lysed in 2× concentrated Laemmli buffer (100 mM Tris/HCl, pH 6.8, 4% SDS, 20% glycerol, 0.05% bromophenol blue, and 5% 2-mercaptoethanol) heated to 95°C. BMDMs and other isolated cell types or cell lines were counted and lysed by adding 2× concentrated Laemmli buffer at 95°C for 5 min. Alternatively, cells were lysed in radioimmunoprecipitation assay buffer (20 mM Tris HCl, pH 7.5, 250 mM NaCl, 10 mM MgCl_2_, 1% NP-40, 0.1% SDS, and 0.5% sodium deoxycholate), supplemented with 5 mM EDTA (Sigma-Aldrich), 1 mM dithiothreitol, complete Mini EDTA free protease inhibitors (Roche), and protease inhibitor cocktail powder (Sigma-Aldrich) and snap-frozen in liquid nitrogen. The samples were centrifuged (4°C, 10,000 *g*, 15 min), and protein concentration was determined by bicinchoninic acid assay (Thermo Fisher Scientific). The samples were denatured with Laemmli for 5 min at 95°C, and equal amounts were loaded for SDS-PAGE. Proteins were separated by SDS-PAGE and blotted to polyvinylidene fluoride (PVDF) membranes (Thermo Fisher Scientific), using standard procedures. 5% milk in TBS was used as blocking reagent.

The following primary antibodies were used: anti-actin (C4; Merck), anti-GAPDH (1A7; in-house), anti–Regnase-3 (4D3; in-house), anti–Regnase-1 (15D11; in-house), anti-p50 (sc-1190; Santa Cruz), anti–β-tubulin (H-235; Santa Cruz), anti-HA (12CA5; in-house), anti-GFP (3H9; in-house), anti-PU.1 (C-3; Santa Cruz), C/EBPα (G-10; Santa Cruz), anti–Elk-1 (9182; Cell Signaling), anti-IRF3 (D83B9; Cell Signaling), and anti-IRF7 (W16064A; BioLegend). Anti-mouse, anti-rat, and anti-rabbit HRP-conjugated secondary antibodies were from Cell Signaling. For chemiluminescence detection, ECL Prime and ECL Select reagents (GE Healthcare) were used, and signals were captured on a LI-COR Odyssey imaging system.

### Regnase-3 protein expression in immune cell types

For B cell isolation, spleens from C57BL/6 mice were cut into pieces and strained through a 70-µm mesh (Falcon), and erythrocytes were lysed in Tris–ammonium chloride buffer. Resuspended splenocytes were labeled with biotinylated anti-CD19 (6D5; BioLegend), and CD19^+^ cells were isolated using anti-biotin MicroBeads (Miltenyi) according to the manufacturer’s protocol. Peripheral CD4^+^ T cells were isolated from the spleen of C57BL/6 mice, using CD4 Dynabeads and DETACHaBEAD (Thermo Fisher Scientific) according to the manufacturer’s protocol and expanded in cell culture. Cell culture plates were coated with goat anti-hamster IgG (MP Biomedicals) to enable soluble anti-CD3 (145-2C11H; in-house) and anti-CD28 (37N; in-house; both from hamster) to bind to the surface of the plate. CD4^+^ T cells were then plated in T cell expansion medium (RPMI 1640, supplemented with 10% FBS [Sigma-Aldrich], 100 U/ml P/S, 0.05 mM β-mercaptoethanol [Life Technologies], 1 mM sodium pyruvate [Life Technologies], 1% GlutaMax solution [Life Technologies], 1% nonessential amino acids solution, 1% MEM vitamin solution, 10 mM Hepes, pH 7.2, 0.1 µg/ml anti-CD3, 1 µg/ml anti-CD28, and 200 IE/ml Proleukin S [IL2-analogue; Novartis]) and expanded for 4 d without further addition of anti-CD3 or anti-CD28. To obtain CD8^+^ T cells, the splenic fraction after depletion of B cells (CD19^+^) and CD4^+^ T cells was expanded for 4 d as described for CD4^+^ T cells.

For preparation of murine BMDMs, bone marrow cells from C57BL/6 mice were strained through a 70-µm mesh (Falcon), and erythrocytes were lysed in Tris–ammonium chloride buffer. Washed cells were resuspended in macrophage differentiation medium (DMEM [Sigma-Aldrich] supplemented with 10% heat-inactivated FBS, 30% L929 supernatant, and 100 U/ml P/S). Cells were plated on non–tissue culture–treated Petri dishes (Greiner) and differentiated for 6 d. On day 3, 50% of the medium was replaced with fresh differentiation medium. On day 6, cells were washed with PBS, dislodged with Accutase (Sigma-Aldrich), and replated in macrophage serum-free medium (Macrophage-SFM; Life Technologies) onto 6-well plates (protein and RNA assays), 96-well plates (phagocytosis assay), or glass coverslips (confocal microscopy).

Bone marrow cells for conventional differentiated DCs (cDCs) were generated as described for BMDMs. Bone marrow cells were resuspended in cDC differentiation medium (IMDM [Sigma-Aldrich] supplemented with 10% heat-inactivated FBS, 1% GlutaMax solution, 100 U/ml P/S, 0.05 mM β-mercaptoethanol, 10 mM Hepes, pH 7.2, 25 ng/ml GM-CSF [Peprotech], and 25 ng/ml IL-4 [Peprotech]), plated on tissue culture–treated plates, and differentiated for 6 d. On day 3, 50% of the medium was replaced by fresh differentiation medium. On day 6, cells were replated into cDC medium without cytokine supplement.

Neutrophils were isolated from bone marrow from C57BL/6 mice using density centrifugation with HistoPaque as described elsewhere (Swamydas et al., 2015). The purity of all cell types was determined by flow cytometry and was between 85 and 98%. The following antibodies were used to determine purity: anti-B220 for B cells, anti-CD4 for CD4^+^ T cells, anti-CD8 for CD8^+^ T cells, anti-F4/80 for BMDMs, anti-CD11c for cDCs, and anti-Gr1 for neutrophils. Cells were counted and lysed in Laemmli buffer and analyzed by immunoblot (see Immunoblotting).

### Blood and serum analysis

Blood was collected from the heart of CO_2_-euthanized *Regnase-3*^+/+^, *Regnase-3*^−/−^, *Regnase-3^fl/fl^*, and *Regnase-3^fl/fl^* +LysM-Cre mice, coagulated, and centrifuged (2,000 *g*, 15 min, 4°C) to obtain blood serum. Plate-bound ELISA was used for quantitative detection of mouse immunoglobulin levels for IgG, IgM, and IgA (all from Affymetrix eBioscience) according to the manufacturer’s protocol and measured on a PHERAstar microplate reader (BMG Labtech). IL-1α, IL-1β, IL-6, IL-10, IL-12p70, IL-17A, IL-23, IL-27, IFNβ, IFNγ, TNFα, and GM-CSF in mouse serum were determined by multiplex assay LEGENDplex mouse inflammation panel (BioLegend) according to the manufacturer’s protocol and analyzed on a FACS AriaIII flow cytometer (BD Biosciences). Peripheral blood counts were analyzed in anticoagulated blood (EDTA) using an automated cell counter, ABX Micros ES60 (Horiba Medical).

### Tissue and nuclear autoreactive antibodies

Mouse serum was prepared as described in Blood and serum analysis. Sera were tested for autoreactive antibodies against tissues as follows. Liver lysates from immunodeficient non-obese diabetic (NOD) scid gamma mice were dissolved by SDS-PAGE and blotted to PVDF membranes, using standard procedures. Membranes were probed with sera from *Regnase-3*^+/+^ and *Regnase-3*^−/−^ mice. Sera from *Rc3h1*^*san/san*^ and *MRL/lpr* mice served as positive controls. Autoreactive IgGs were visualized using an anti-mouse HRP-conjugated secondary antibody (Cell Signaling) and by chemiluminescence on photo films. Samples were scored as 0 = negative, 1 = weak positive, 2 = strong positive. For detection of antinuclear antibodies, immobilized HEp-2 cells (Euroimmun) were probed with sera from *Regnase-3*^+/+^ and *Regnase-3*^−/−^ mice or MRL/lpr mice (positive control). Autoreactive IgG were visualized using FITC-coupled anti-mouse IgG (sc-2010; Santa Cruz). Images were captured by a BZ-9000 microscope (Keyence) and scored as 0 = negative, 1 = weak positive, 2 = strong positive.

### Confocal microscopy

BMDMs from C57BL/6J mice were differentiated as described in Regnase-3 protein expression in immune cell types. For immunofluorescence, 4 × 10^5^ cells were seeded on glass coverslips (VWR) in Macrophage-SFM (Life Technologies). The next day, cells were pretreated for 4 h with 10 µg/ml high molecular weight poly-I:C (InvivoGen) or 25 µM chloroquine (Sigma-Aldrich) or were left untreated. If indicated, cells were treated with lysine-fixable Texas Red–coupled dextrane molecules (molecular weight, 10,000 daltons; Thermo Fisher Scientific) or Lysotracker LYSO-ID red lysosomal detection kit (Enzo) for 0.5 h each. HEK293T cells were transfected with *GFP-Regnase-1*, *GFP-Regnase-3*, *Flag-Regnase-1* (all in pLNCX2), or *GFP-Roquin-1* (in pDEST12.2) as indicated, using the calcium phosphate method. For immunofluorescence, 2.5 × 10^5^ HEK293T cells were seeded on poly-l-lysine–coated glass coverslips.

BMDM and HEK293T cells were fixed for 10 min in 4% Roti-Histofix (Roth) at RT, washed (PBS with 0.1% Tween-20), and permeabilized for 15 min in permeabilization buffer (PBS with 0.1 M glycin and 0.1% Triton X-100). Cells were then washed, blocked for 1 h in blocking solution (PBS with 0.1% Tween-20, 10% FBS, 0.1% BSA, and 3% donkey serum), and probed with primary antibodies in blocking solution at 4°C overnight. Cells were washed three times, probed with fluorophore-labeled secondary antibodies in blocking solution for 1 h at room temperature, and washed twice. Nuclei were counterstained with DAPI in PBS and washed twice in PBS, and coverslips were mounted on SuperFrost slides (Thermo Fisher Scientific) in mounting medium (2.5% 1,4-diazabicyclo-octane, 10% Mowiol 4-88, 25% glycerol, and 0.1 M Tris-HCl) and sealed. Slides were analyzed on a Leica SP5 confocal laser scanning microscope. The primary antibodies used were anti–Regnase-3 (3D4; in-house), anti-DDX6 (rabbit, polyclonal, A300-461A; Bethyl), anti-EEA1 (C45B10; Cell Signaling), anti-Rab7 (D95F2; Cell Signaling), anti-Rab11 (D4F5; Cell Signaling), anti–Clathrin heavy chain (D3C6; Cell Signaling), anti-LC3B (2H30L32; Life Technologies), and anti–FLAG-tag (6F7; in-house). The secondary antibodies used were Cy5-anti-rabbit IgG, Cy5-anti-mouse IgG, AF488-anti rat IgG, Cy3-anti-rat IgG, and Cy5-anti-rat IgG (all from Jackson ImmunoResearch).

### *Regnase* mRNA expression levels in immune cell subsets

BMDMs and cDCs were generated as described in Regnase-3 protein expression in immune cell types and stimulated as described below. CD4^+^ T cells were isolated from the spleens of C57BL/6 wild type mice using CD4 Dynabeads and DETACHaBEAD (Thermo Fisher Scientific) according to the manufacturer’s protocol and stimulated as described below. CD8^+^ T cells and B220^+^ B cells were isolated from the spleens of C57BL/6 wild type mice, using positive selection MicroBeads (Miltenyi) for B220 and CD8, respectively. BMDMs and cDCs were stimulated with 100 ng/ml LPS (Sigma-Aldrich), 20 µg/ml low molecular weight poly-I:C (Merck), and 1 µM C-type CpG oligodeoxynucleotide (ODN) 2395 (Miltenyi) for 1, 4, and 16 h or were left untreated. B cells and CD4^+^ and CD8^+^ T cells were resuspended in lymphocyte medium (RPMI 1640 [Life Technologies], supplemented with 10% FBS [Life Technologies], 100 U/ml P/S [Life Technologies], 0.05 mM β-mercaptoethanol [Life Technologies], 1 mM sodium pyruvate [Life Technologies], 1% GlutaMax solution [Life Technologies], 1% nonessential amino acids solution [Life Technologies], 1% MEM vitamin solution [Life Technologies], and 10 mM Hepes, pH 7.2 [Life Technologies]), and stimulated as follows. B cells were treated with 10 µg/ml anti-IgM (HB88; in-house), 20 µg/ml LPS (Sigma-Aldrich), or 1 µM C-type CpG ODN 2395 (Miltenyi) for 1, 4, and 16 h or were left untreated. T cell receptor signaling in CD4^+^ and CD8^+^ T cells was activated with plate-bound anti-CD3/CD28 as follows. Cell culture plates were coated with goat anti-hamster IgG (MP Biomedicals) to enable soluble anti-CD3 (145-2C11H; in-house) and anti-CD28 (37N; in-house; both from hamster) to bind to the surface of the plate. T cells were activated with 1 µg/ml anti-CD3 and 2.5 µg/ml anti-CD28. Alternatively, T cells were treated with 20 µg/ml low molecular weight poly-I:C (Merck). T cells were stimulated for 1, 4, and 16 h or were left untreated. Immune cells were then lysed with Trizol lysis reagent (Qiagen) and processed as described in Real-time RT-PCR.

### Regnase-3 antibody generation and characterization

To generate an antibody against murine Regnase-3 protein, aa 1–903 of the Regnase-3 protein sequence with N-terminal GST-tag and C-terminal His-tag were expressed in *Escherichia coli* and purified in two steps on a HiTrap Chelating and a GSTrap column. Lou/C rats and C57BL/6 mice were immunized with the Regnase-3 peptide as described elsewhere (Feederle et al., 2016). Specific antibodies were first selected by enzyme-linked immunoassay on the protein and, in a second step, by immunoblotting using GFP-Regnase-3 protein lysates, expressed in HEK293T cells. Antibodies were finally verified by immunoblotting of overexpression lysates from all Regnase family members as well as in *Reganse-3*^+/+^ and *Regnase-3*^−/−^ protein lysates. The hybridoma cells of Regnase-3–reactive supernatants were cloned at least twice by limiting dilution. Rat monoclonal antibody 4D3 (IgG2a) was used in this study. Protein alignment of Regnase family members was done using T coffee (http://tcoffee.crg.cat) with the M-Coffee algorithm.

### Classical and alternative activation of BMDMs (M1/M2)

BMDMs were generated from C57BL/6 mice as described in Regnase-3 protein expression in immune cell types. BMDMs were classically activated with 100 ng/ml LPS from *S. enterica* serotype typhimurium together with 20 ng/ml IFNγ (R&D Systems; M1 condition), alternatively activated with 20 ng/ml IL-4 (R&D Systems; M2 condition) for 24 h, or were left untreated (M0 condition) and analyzed by immunoblot. Shifts toward M1 and M2 condition were verified with surface markers reported to be up-regulated under M1 condition (CD38) or M2 condition (CD206) by flow cytometry analysis.

### Stimulation of BMDMs for immunoblot and RT-PCR analysis

BMDMs from C57BL/6J, *Regnase-3^+/+^*, and *Regnase-3*^−/−^ or *NF-κB p50*^−/−^ mice were differentiated as described in Regnase-3 protein expression in immune cell types, and 10^6^ cells were plated in Macrophage-SFM on non–tissue culture treated 6-well dishes (Falcon). Cells were preincubated with 10 µM (S)-MG132 (Cayman Chemical) or DMSO (vehicle) if indicated, or were left untreated. Cells were then stimulated with 100 ng/ml LPS from *S. enterica* serotype typhimurium (Sigma-Aldrich), 1 µg/ml lipoteichoic acid (LTA) from *S. aureus* (Sigma-Aldrich), 1 µg/ml R848 (Resiquimod; Cayman Chemical), 100 ng/ml ultrapure Flagellin (InvivoGen), 10 µg/ml high molecular weight poly-I:C (Invivogen), 1 µg/ml C-type CpG ODN 2395 (Miltenyi), 100 ng/ml Murabutide (InvivoGen), 25 ng/ml PMA (Sigma-Aldrich), 50 ng/ml TNFα (Novus Biologicals), 50 ng/ml IL-1β (R&D Systems), 50 ng/ml IL-4 (R&D Systems), 50 ng/ml IL-6 (R&D Systems), 50 ng/ml IL-10 (R&D Systems), 50 ng/ml IL-15 (R&D Systems), 50 ng/ml IFNγ (R&D Systems), or 50 ng/ml IFNγ plus 100 ng/ml LPS for the indicated times. For analysis of TLR3 downstream signaling, BMDMs were preincubated for 5 h with 20 µM Ikkε/TBK1 inhibitor MRT67307 (Merck) or DMSO (vehicle) and stimulated with 10 µg/ml high molecular weight poly-I:C for 1 and 4 h or were left untreated. For immunoblot analysis, BMDMs were lysed by adding 2× concentrated Laemmli buffer at 95°C; for RNA analysis, cells were lysed in Trizol reagent and processed as described in Immunoblotting and Real-time RT-PCR.

### RNA sequencing of B cells and alveolar macrophages

Alveolar macrophages were isolated by bronchoalveolar lavage with five rinsing steps each of 1 ml cold (4°C) rinsing buffer (PBS, 2.5 mM EDTA, and 2.5% FBS) through 21G catheter tubing (B. Braun). Fc receptors were blocked using anti-CD16/32 (FCR3-4G8; in-house) before staining with anti–CD11c-biotin and anti-CD11c-APC (N418; BioLegend). Addition of CD11c-APC allows subsequent verification of the isolation efficiency by flow cytometry. Alveolar macrophages were then positively selected using anti-biotin microbeads (Miltenyi). Cells were lysed in buffer RLT Plus (Qiagen), and RNA was isolated according to the manufacturer’s protocol.

For B cell isolation, lymph nodes were dissected from *Regnase-3^+/+^* and *Regnase-3*^−/−^ mice and divided into normal-sized and enlarged lymph nodes in each *Regnase-3*^−/−^ mouse. CD19^+^ B cells were isolated using biotinylated anti-CD19 (6D5; BioLegend), and CD19^+^ cells were isolated using anti-biotin MicroBeads (Miltenyi) according to the manufacturer’s protocol. Cells were lysed in Trizol reagent, and total RNA was isolated according to the manufacturer’s protocol.

B cell and alveolar macrophage RNA was sequenced as follows. Library preparation and rRNA depletion were performed using the TruSeq Stranded Total RNA Library Prep Kit (Illumina). Barcoded B cell libraries were sequenced on a HiSeq 2500 (Illumina) with paired-end, 100-bp reads, and barcoded alveolar macrophage libraries were sequenced on a HiSeq 4000 (Illumina) with paired-end, 150-bp reads. For B cell samples, reads were mapped against the mouse genome mm9 (UCSC version mm9, NCBI37) using STAR. To quantify the number of reads mapped to annotated genes, we used the featureCounts program from the subread package (v1.6.0; Liao et al., 2014). To identify differentially expressed genes, DESeq2 was employed (Varet et al., 2016). Gene ontology (GO) analysis was performed using Gorilla (Eden et al., 2009).

For alveolar macrophage samples, reads were mapped against the mouse genome mm10 (GRCm38) using HISAT2. To quantify the number of reads mapped to annotated genes, we used the feature Counts program from the subread package. To identify differentially expressed genes, edgeR was employed (Robinson et al., 2010). GO analysis was performed using Enrichr (Kuleshov et al., 2016). Redundant GO terms were removed using REViGO (Supek et al., 2011). The RNA sequencing data were deposited in the Gene Expression Omnibus database under accession no. GSE129325.

### RNA and poly-I:C electrophoretic mobility shift assays (EMSAs) and degradation assays

High molecular weight poly-I:C was purchased from InvivoGen. INFγ, Regnase-3, Regnase-1, and HPRT 3′ UTR RNAs were in vitro transcribed with in-house–produced T7 polymerase. Briefly, the transcription reaction (600 ng DNA template [PCR subcloned from plasmids used in luciferase assays to contain the T7 promoter region]; 20–40 mM MgCl_2_; 10% PEG 8000; 8 mM each rATP, rCTP, rGTP, and rUTP; 40 mM Tris, pH 8; 1 mM spermidine; 0.01% Triton X-100; 5 mM dithiothreitol; and 0.06 mg/ml T7 polymerase) was incubated at 37°C for 3 h. The RNAs were purified under denaturing conditions using anion exchange HPLC, followed by dialysis against a high (1 M) to low (0.1 M) NaCl solution gradient. Following exchange in 0.1 M NaCl, the RNAs were dialyzed against ddH_2_O. The RNAs were concentrated to 0.3 mg/ml using a 10-kD molecular mass cutoff Amicon concentrator. The concentrations were measured using NanoDrop.

In vitro binding and cleavage assays were performed in buffer containing 50 mM Tris, pH 8, 100 mM NaCl, 5 mM MgCl_2_, 1 mM dithiothreitol, and 6.5 mM ZnCl_2_. RNAs were incubated with varying concentrations (ranging from 0.2 to 23 µM) of Regnase-3 aa 1–478 and Regnase-3 mut and allowed to incubate at room temperature for 4 h. Positive degradation controls with RNase T1, RNase A, or S1 nuclease (New England Biolabs) were performed alongside binding and cleavage assays. For binding assays, the reaction mixtures were supplemented with 10% glycerol and loaded onto a 1% agarose gel prepared with 1× Tris-acetate-EDTA buffer and stained with Serva DNA Stain Clear G. Gels were run at 100 V for 25 min at room temperature in 1× Tris-acetate-EDTA running buffer. For cleavage assays, reactions were stopped with the addition of denaturing 6× loading dye (8 M urea, 0.02 M EDTA, 0.002 M Tris, pH 7.5, 0.025% [wt/vol] bromophenol blue, and 0.025% [wt/vol] xylene cyanol) followed by heat denaturing at 95°C for 5 min. RNAs were immediately loaded onto a 30% (poly-I:C and other small RNAs [<20 nucleotides]) or 12.5% (Regnase-1 3′UTR RNA) denaturing polyacrylamide gel and allowed to run for 2 h (15 W) at room temperature. The polyacrylamide gels were stained with SYBR Gold (Thermo Fisher Scientific) for 5 min before imaging.

### Cytokine ELISA in BMDM supernatants

BMDMs were differentiated and cultivated as described in Regnase-3 protein expression in immune cell types. On the last day of differentiation, BMDMs (10^5^) were plated in 96-well flat bottom plates. The next day, cells were stimulated for the indicated times. BMDMs were stimulated with LPS (200 ng/ml; InvivoGen), Pam3CSK4 (2 µg/ml; InvivoGen), R848 (2 µg/ml; InvivoGen), or poly-I:C (10 µg/ml; InvivoGen) for 8 h and 24 h. Additionally, 1 µg/ml of herring testes DNA (HT-dsDNA; Sigma-Aldrich), poly-I:C, or 5′ triphosphate dsRNA (5′ppp-RNA) was complexed to Lipofectamine 2000 (Life Technologies) according to the manufacturer’s protocol, and cells were transfected with the mix for 8 h and 24 h. Supernatants were taken for TNF, IL-6 (both BD Bioscience), and IP-10 (CXCL-10; R&D Systems) ELISA. ELISAs were performed according to the manufacturer’s instructions.

### Phagocytosis in BMDMs

BMDMs from *Regnase-3^+/+^* and *Regnase-3*^−/−^ mice were differentiated as described in Regnase-3 protein expression in immune cell types, and 1.5 × 10^5^ cells/well were plated in Macrophage-SFM on 96-well plates. BMDMs were left untreated or were preincubated for 4 h with 100 ng/ml LPS from *S. enterica* serotype typhimurium (Sigma-Aldrich), 1 µg/ml LTA from *S. aureus* (Sigma-Aldrich), 10 µg/ml high molecular weight poly-I:C (InvivoGen), or 50 ng/ml TNFα (Novus Biologicals). Cells were then probed with *S. aureus* bioparticles coupled to pH-sensitive pHrodo green fluorophore (Invitrogen) and centrifuged for 1 min at 200 *g*. The increase in fluorescence was acquired over time with a VarioSkan Lux microplate reader (Thermo Fisher Scientific).

### Mouse pneumonia model

Methicillin-resistant *S. aureus* (MRSA, strain USA300) was cultured in brain–heart infusion (BHI; Bacto Brain Heart Infusion [Porcine]; BD) medium at 37°C with 180 rpm shaking. The CFUs were determined for OD_600_ = 0.1 with 2 × 10^8^ CFU/ml. For infection experiments, *S. aureus* was inoculated in BHI medium (37°C, 180 rpm) overnight. The overnight culture was diluted in BHI to OD_600_ = 0.1 and further incubated at 37°C, 180 rpm for 1.5 h. Subsequently, OD_600_ was measured and adjusted to OD_600_ = 0.25, which equals 5 × 10^8^ CFU/ml. 1 ml bacterial culture OD_600_ = 0.25 was centrifuged at 6,000 *g* for 10 min. The supernatant was discarded, and the pellet was resuspended in 20 µl of 0.9% sodium chloride solution (Braun) and kept on ice until injection. Mice were anesthetized by i.p. injection of 5 mg/kg midazolam, 0.05 mg/kg fentanyl, and 0.5 mg/kg medetomidine hydrochloride and then inoculated intranasally with MRSA suspension (5 × 10^8^ CFUs in 20 µl). 24 h after bacterial infection, mice were sacrificed, and lung tissue was removed for quantification of bacteria and histological analysis.

The right lungs were homogenized using a TissueLyser (Qiagen) with an oscillation of 50/s for 10 min. Serial dilutions were made in BHI and plated on MRSA II plates (Oxoid; Thermo Fisher Scientific). Plates were incubated at 37°C for 20 h. Colonies were counted, and the CFU/g tissue was calculated. For immunofluorescence staining, part of the left lung was fixed in 4% paraformaldehyde for 2 h, placed in sucrose 30% for 12 h, and then embedded in optimal cutting temperature medium. Frozen tissue samples were cut with a cryotome (CryoStar NX70; Thermo Fisher Scientific) into 10-µm sections and blocked with 5% serum. The sections were incubated with primary antibodies (1:1,000) against *S. aureus* (rabbit polyclonal, ab20920; Abcam) and macrophages (CD68, clone FA-11, MCA1957; Bio-Rad) followed by incubation with AF488- and AF555-conjugated secondary antibodies (1:2,000; Invitrogen). DNA was stained with 1 µg/ml DAPI (28718-90-3; Sigma-Aldrich). Images were acquired using a LSM 880 confocal microscope with Airyscan module and Plan-Apochromat ×20/0.8 air objective (Zeiss) and processed using ZEN software (Zeiss). Animal studies for the mouse pneumonia model were approved by the government of Bavaria (AZ 55.2-1-54-2532-190-2015 and AZ 55.2-1-54-2532-215-2014).

### Statistical analysis

Data analysis was performed using Prism software (GraphPad) and the indicated test; *, P ≤ 0.05; **, P ≤ 0.01; ***, P ≤ 0.001; ****, P ≤ 0.0001; and ns, not significant.

### Online supplemental material

Fig. S1 describes generation of Regnase-3–deficient mice, survival rates, and calculated total cell numbers in lymph nodes. Fig. S2 shows additional bioinformatics analyses for RNA sequencing of B cells and flow cytometry blots and statistics for immune cell subsets of Regnase-3–deficient mice. Fig. S3 shows Regnase family member mRNA expression levels in tissues and immune cells, Regnase-3 antibody verification, calculated total cell numbers in lymph nodes of *Regnase-3^fl/fl^* +LysM-Cre mice, Hoxb8 myeloid progenitor cell differentiation, and M1/M2 activation of macrophages. Fig. S4 shows immunoblots for the analysis of Regnase-1 and -3 protein in response to cytokines and TLR agonists in wild type cells and cells deficient for *NEMO*, *IRF3*, *IRF7*, or *NF-κB*. Fig. S5 shows subcellular localization of Regnase-3 and effects of Regnase-3 on phagocytosis in vitro and in vivo.

## Supplementary Material

Supplemental Materials (PDF)
