# Supplementary material for: Immune homeostasis and regulation of the interferon pathway require myeloid-derived Regnase-3
Source: J Exp Med. 2019 May 24;216(7):1700–23. doi: 10.1084/jem.20181762 (PMC6605757; doi:10.1084/jem.20181762)
Supplement: Supplemental Materials (PDF) [file JEM_20181762_sm.pdf]

Supplemental material

von Gamm et al., https://doi.org/10.1084/jem.20181762

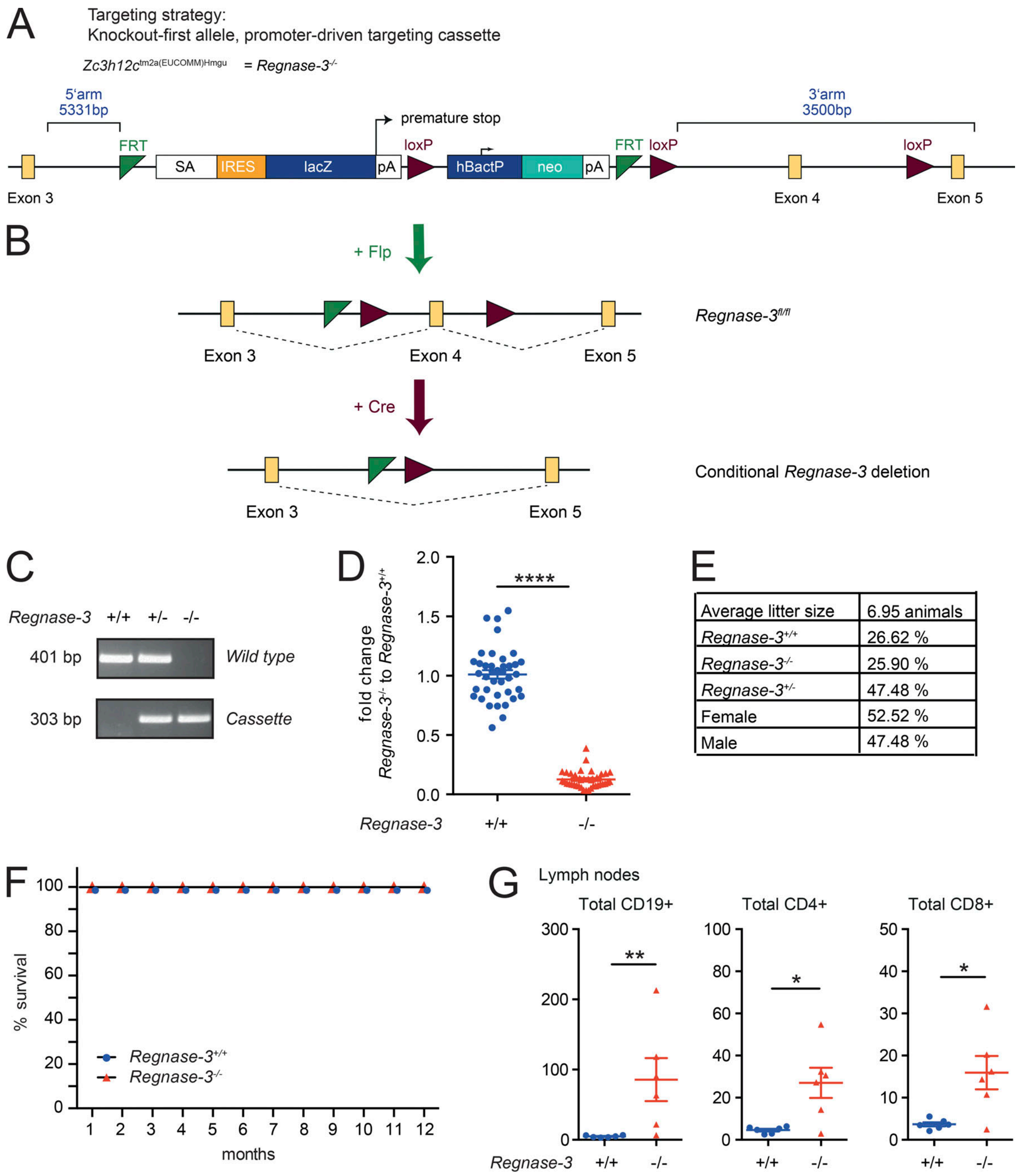

Figure S1. **Generation of *Regnase-3* (*Zc3h12c*)-deficient mice. (A)** Schematic representation of the targeting vector for generation of *Regnase-3* (*Zc3h12c*)-deficient mice: FRT, FLP recombinase target; SA, splice acceptor; IRES, internal ribosome entry site; lacZ, β-galactosidase; pA, polyA; hBactP, promoter; neo, neomycin resistance gene. **(B)** Mating strategies for generation of mice with conditional deletion of *Regnase-3* (*Zc3h12c*) with promoter-driven Cre recombinases. **(C)** PCR analysis of mouse ear tissue to identify the *Regnase-3* genotype using wild type primers and primers to identify the cassette. **(D)** Quantitative RT-PCR of the *Regnase-3* (*Zc3h12c*) gene transcript in liver tissue from *Regnase-3*$^{+/+}$ and *Regnase-3*$^{-/-}$ mice (n = 39/39), relative to expression in *Regnase-3*$^{+/+}$ mice. **(E)** 20 representative litters from het × het matings with a total of 139 mice were used to calculate average litter size and the distribution of the genotypes and sex. **(F)** Survival rate in a cohort of *Regnase-3*$^{+/+}$ and *Regnase-3*$^{-/-}$ mice (n = 10/10). **(G)** Number of calculated total B cells (CD19$^+$) and CD4$^+$ and CD8$^+$ T cells in lymph nodes of *Regnase-3*$^{+/+}$ and *Regnase-3*$^{-/-}$ mice (n = 6/6). Data are represented as mean ± SEM and were compared by Mann–Whitney *U* test (*, $P \leq 0.05$; **, $P \leq 0.01$; ****, $P \leq 0.0001$).

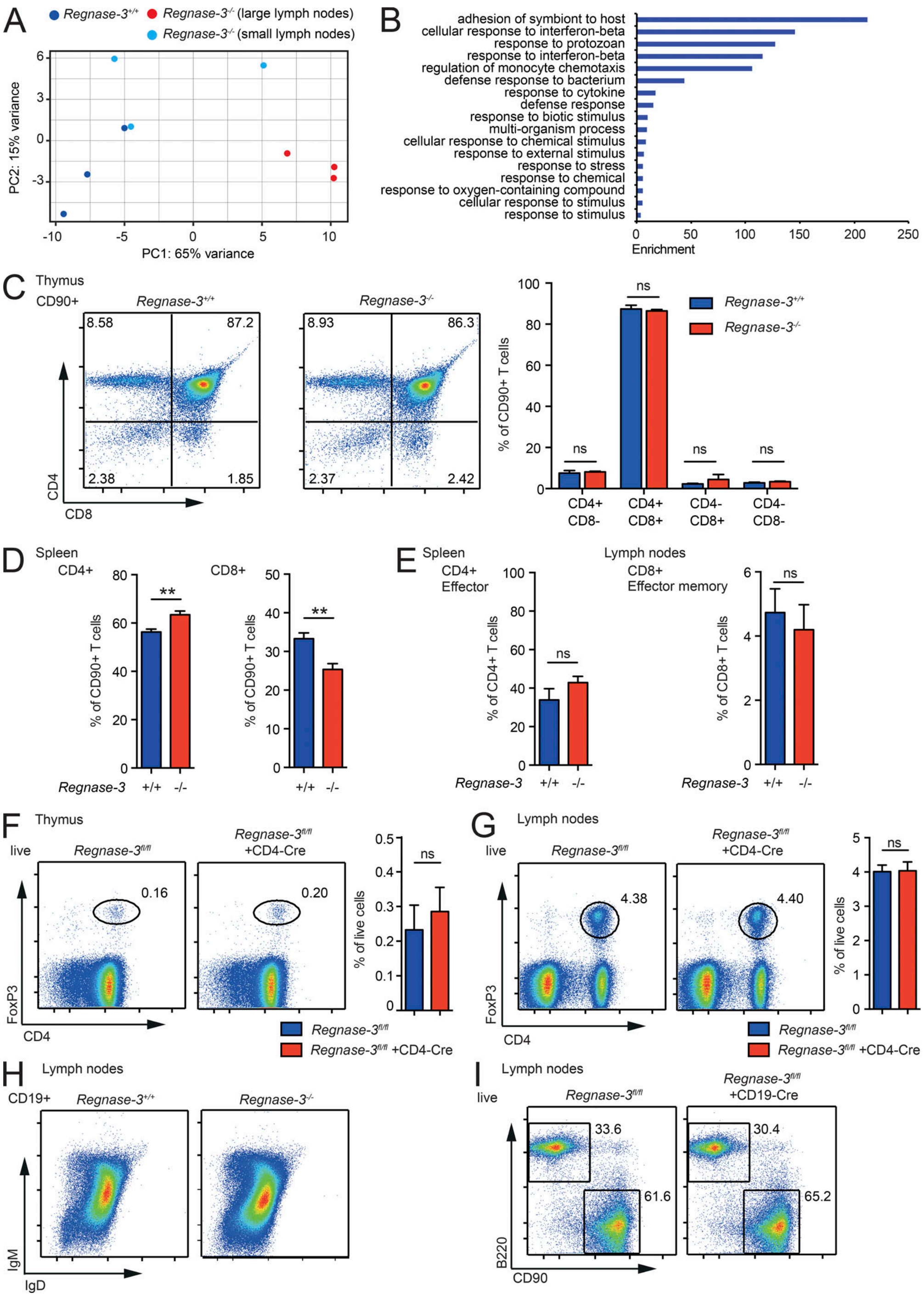

Figure S2. **T and B cell analysis of *Regnase-3*$^{-/-}$ mice. (A)** B cells (CD19$^+$) were isolated from enlarged lymph nodes and normal-sized lymph nodes from the same *Regnase-3*$^{-/-}$ mice and their *Regnase-3*$^{+/+}$ littermates (n = 3/3), and RNA was isolated and subjected to RNA sequencing. The individual B cell samples are plotted based on PCA. **(B)** Enriched GO terms (P < 0.05) for all up-regulated (≥2 log2 fold) genes (as shown in Fig. 4) ordered according to their enrichment value. **(C)** Frequencies of double-negative, double-positive, and CD4 and CD8 single-positive T cells in the thymus of *Regnase-3*$^{-/-}$ mice and their *Regnase-3*$^{+/+}$ littermate controls at 6 mo of age assessed by flow cytometry (n = 8/8). Left: Representative blots. Right: Statistics. **(D)** Frequencies of splenic CD4$^+$ and CD8$^+$ T cells within the T cell compartment (CD90$^+$) of *Regnase-3*$^{-/-}$ mice and their *Regnase-3*$^{+/+}$ littermate controls at 6 mo of age assessed by flow cytometry (n = 9/9). **(E)** Frequencies of splenic CD4$^+$ effector T cells (CD19$^-$, CD90$^+$, CD4$^+$, CD62L$^-$/CD44^{hi}) in *Regnase-3*$^{-/-}$ mice and their *Regnase-3*$^{+/+}$ littermate controls at 6 mo of age (n = 6/6), and frequencies of CD8$^+$ effector memory T cells (CD19$^-$, CD90$^+$, CD8$^+$, CD62L$^-$/CD44^{hi}) in lymph nodes of *Regnase-3*$^{-/-}$ mice and their *Regnase-3*$^{+/+}$ littermate controls at 6 mo of age (n = 6/6). **(F and G)** Frequencies of regulatory T cells (CD4$^+$, FoxP3$^+$) in *Regnase-3*$^{fl/fl}$ +CD4Cre mice and *Regnase-3*$^{fl/fl}$ controls at 5 mo of age in the thymus (F) and lymph nodes (G), assessed by flow cytometry (n = 9/9). Left: Representative blots. Right: Statistics. **(H)** IgD versus IgM within B cells (CD19$^+$) in lymph nodes of *Regnase-3*$^{-/-}$ mice and their *Regnase-3*$^{+/+}$ littermate controls at 6 mo of age, assessed by flow cytometry (representative blot of n = 6/6). **(I)** Frequencies of B cells (B220$^+$) and T cells (CD90$^+$) in lymph nodes of *Regnase-3*$^{fl/fl}$ +CD19-Cre mice and their *Regnase-3*$^{fl/fl}$ littermates at 5 mo of age (representative flow cytometry blots of n = 13/13). Data are represented as mean ± SEM and were compared by Mann–Whitney U test (**, P ≤ 0.01; ns, not significant).

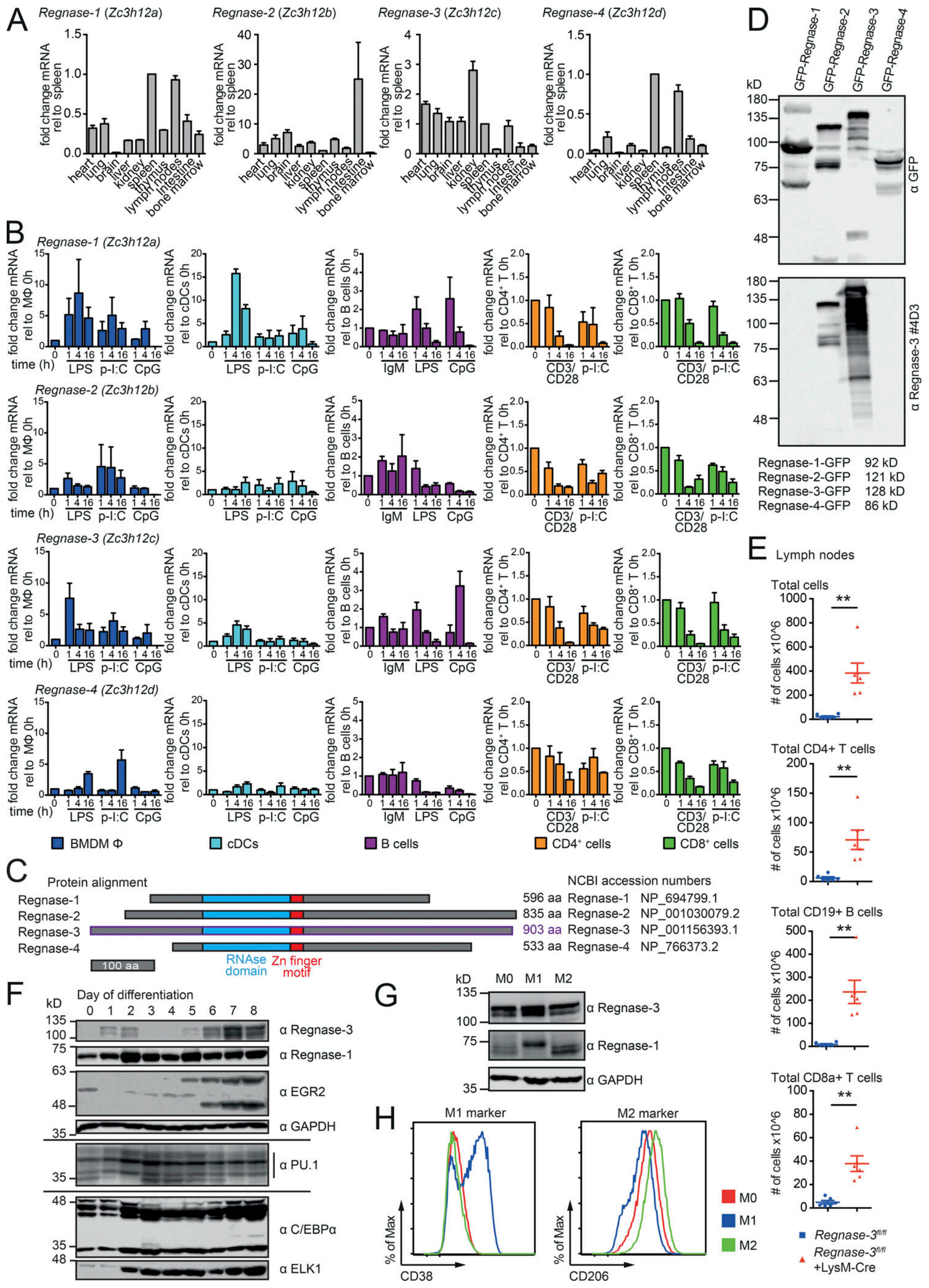

Figure S3. ***Regnase*** **family member mRNA expression levels in tissues and immune cells. (A)** mRNA expression in tissues for *Regnase-1* (*Zc3h12a*), *Regnase-2* (*Zc3h12b*), *Regnase-3* (*Zc3h12c*), and *Regnase-4* (*Zc3h12d*) by quantitative RT-PCR, normalized to *Hprt* relative to (relto) their expression in spleen. Mean ± SEM from three C57BL/6J mice. **(B)** mRNA expression in immune cells for *Regnase1-4* (*Zc3h12a-d*) by quantitative RT-PCR, normalized to *Hprt* and relative to untreated cells. Mean ± SEM from three independent experiments: In vitro differentiated BMDMs (Φ) and in vitro differentiated cDCs were treated with LPS (100 ng/ml), low molecular weight poly-I:C (20 µg/ml), or C-type CpG ODN 2395 (1 µM) for indicated times. Splenic B cells (B220⁺) were stimulated with anti-IgM (10 µg/ml), LPS (20 µg/ml), or C-type CpG ODN 2395 (1 µM) for indicated times. Splenic CD4⁺ and CD8⁺ T cells were stimulated with plate-bound anti-CD3 and anti-CD28 or low molecular weight poly-I:C (20 µg/ml) for indicated times. **(C)** Protein alignment of Regnase family members (National Center for Biotechnology Information accession nos. as indicated). NCBI, National Center for Biotechnology Information. **(D)** Verification of anti–Regnase-3 antibody (clone 4D3): Regnase family members with N-terminal GFP tag were overexpressed and analyzed by immunoblot. Top: Anti-GFP as loading control. Bottom: Anti–Regnase-3 antibody (clone 4D3). **(E)** Number of total cells and calculated total B cells (CD19⁺) and CD4⁺ and CD8⁺ T cells in lymph nodes of Regnase-3$^{fl/fl}$ +LysM-Cre mice and their Regnase-3$^{fl/fl}$ littermate controls at 6 mo of age (*n* = 6/6). **, P ≤ 0.01. **(F)** ER-Hoxb8 cells were differentiated into macrophages by estrogen withdrawal and addition of recombinant GM-CSF (20 ng/ml). Cells were harvested after each day of differentiation and analyzed by immunoblot (representative blot from *n* = 2 independent experiments). **(G)** BMDM from C57BL/6J mice were classically activated (M1 condition) with LPS (100 ng/ml) and IFNγ (20 ng/ml), alternatively activated (M2 condition) with IL-4 (20 ng/ml), or left untreated (M0 condition) for 24 h and analyzed by immunoblot (representative blot of three experiments). **(H)** Shift toward M1 and M2 condition in G was verified with surface markers reported to be up-regulated under M1 condition (CD38) or M2 condition (CD206) by flow cytometry.

https://doi.org/10.1084/jem.20181762

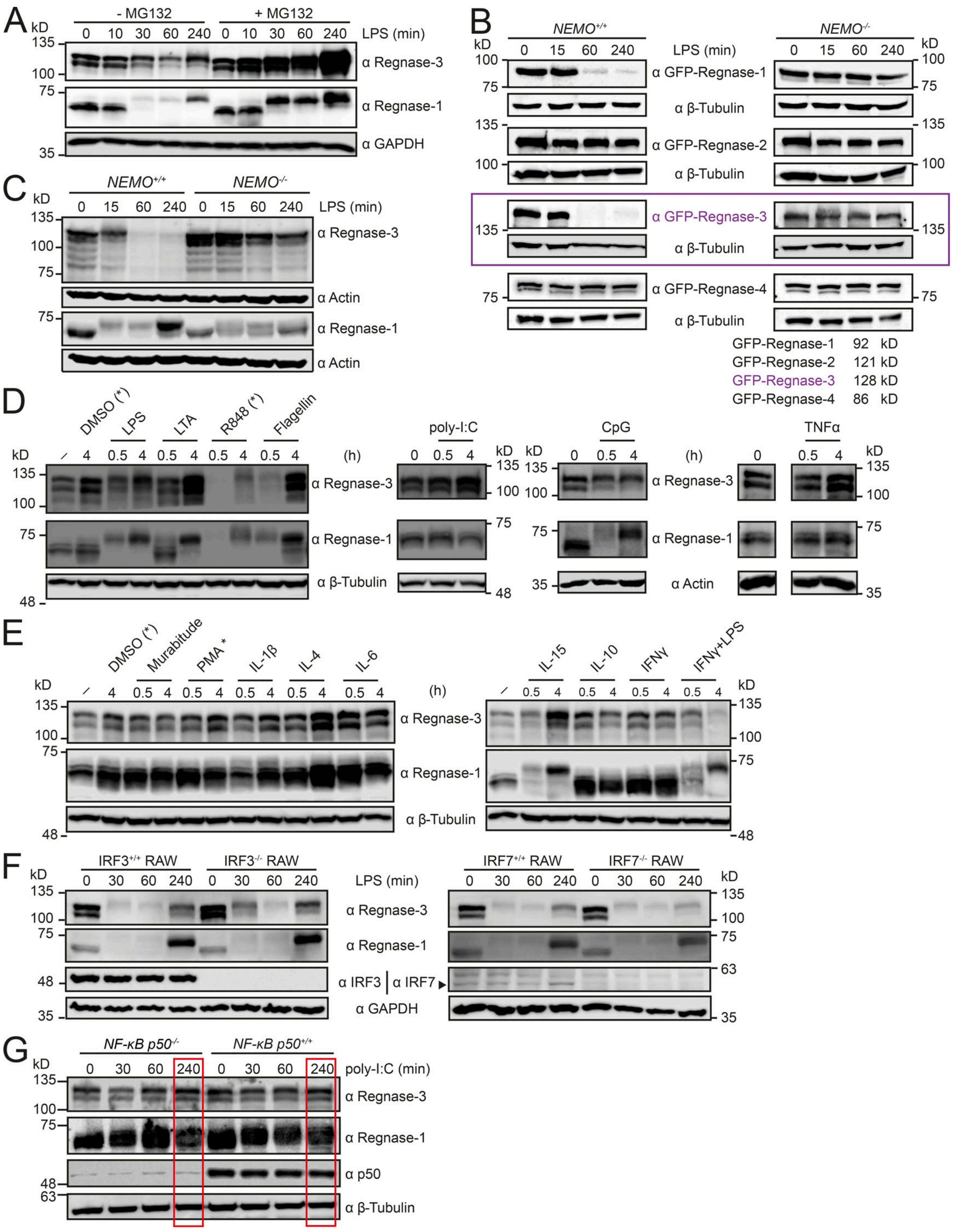

Figure S4. **Regulation of Regnase-1 and -3 protein levels in the presence of cytokines and TLR agonists. (A)** BMDMs from C57BL/6J mice were pre-incubated with MG132 (10 µM) or DMSO (vehicle) for 30 min and then stimulated with LPS (100 ng/ml) for indicated times and analyzed by immunoblot (representative blot of three independent experiments). **(B)** Constructs of Regnase family members Regnase-1–4 with N-terminal GFP tag were overexpressed in *NEMO*-deficient MEFs or *NEMO*-reconstituted cells, stimulated with LPS (1 µg/ml) for indicated times, and analyzed by immunoblot (representative blot of three independent experiments). **(C)** *NEMO*-deficient MEFs or *NEMO*-reconstituted cells were stimulated with LPS (1 µg/ml) for indicated timepoints and analyzed by immunoblot (representative blot of three independent experiments). **(D and E)** BMDMs from C57BL/6J mice were left untreated, were treated with vehicle (compounds dissolved in DMSO are marked with asterisk), or were stimulated with LPS (100 ng/ml), LTA (1 µg/ml), R848 (Resiquimod; 1 µg/ml), Flagellin (100 ng/ml), high molecular weight poly-I:C (10 µg/ml), C-type CpG ODN 2395 (1 µg/ml), TNFα (50 ng/ml), Murabutide (100 ng/ml), PMA (25 ng/ml), IL-1β (50 ng/ml), IL-4 (50 ng/ml), IL-6 (50 ng/ml), IL-15 (50 ng/ml), IL-10 (50 ng/ml), IFNγ (50 ng/ml), or IFNγ plus LPS (50 and 100 ng/ml) for 0.5 or 4 h and analyzed by immunoblot (representative blots of three experiments). **(F)** *IRF3*- or *IRF7*-deficient RAW cells and wild type RAW cells were stimulated with LPS (100 ng/ml) for indicated times and analyzed by immunoblot (representative blot of three independent experiments). **(G)** BMDMs from *NF-κB p50*$^{+/+}$ and *NF-κB p50*$^{-/-}$ mice were stimulated with high molecular weight poly-I:C (µg/ml) for indicated times and analyzed by immunoblot (representative blot from two mice).

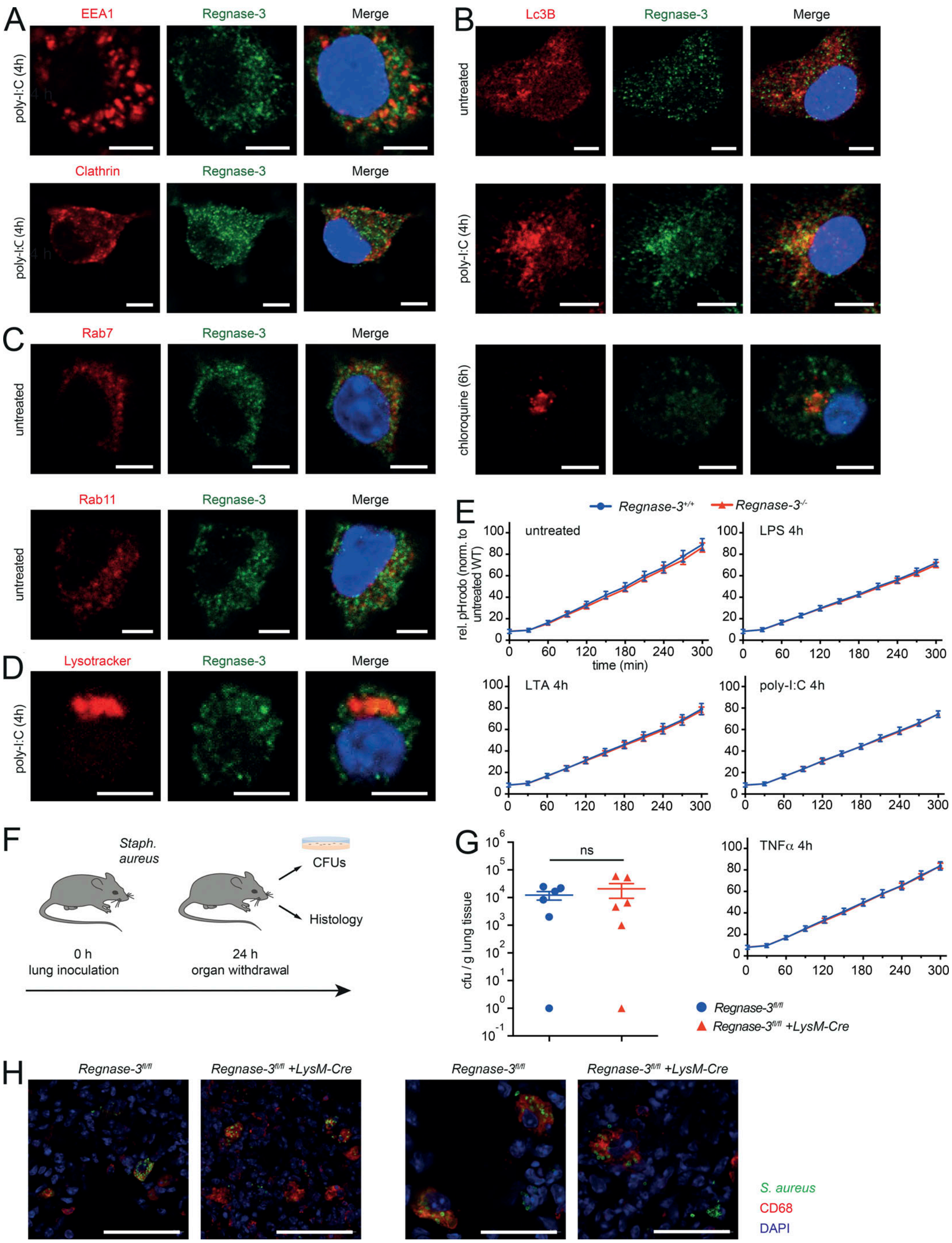

Figure S5. **Subcellular localization and effects of Regnase-3 on phagocytosis. (A–D)** Confocal microscopy images. BMDMs from C57BL/6J mice were pretreated with high molecular weight poly-I:C (10 µg/ml) for 4 h, autophagy inhibitor chloroquine (25 µM) for 6 h, or left untreated, as indicated. Red and green as indicated; counterstain for nuclei with DAPI in blue. Representative images of three or more independent experiments are shown. Bars, 5 µm. **(A)** BMDMs from C57BL/6J mice were immunostained for Regnase-3 and EEA1 (early endosomes) or Clathrin (Clathrin-mediated endocytosis). **(B)** Immunostaining for autophagosome marker Lc3B and Regnase-3. **(C)** Immunostaining for Regnase-3 and endosomal markers Rab7 or Rab11. **(D)** Lysosomes in poly-I:C–treated BMDMs were labeled with LysoTracker dye for 30 min, fixed, and immunostained for Regnase-3. **(E)** BMDMs from *Regnase-3*$^{+/+}$ and *Regnase-3*$^{-/-}$ mice were left untreated or were preincubated for 4 h with LPS (100 ng/ml), LTA (1 µg/ml), high molecular weight poly-I:C (10 µg/ml), or TNFα (50 ng/ml). Subsequently, *S. aureus* bioparticles coupled to pH-sensitive pHrodo fluorophore were subjected to cells, and the increase in fluorescence was acquired over time with a microplate reader. Maximal fluorescence of unstimulated *Regnase-3*$^{+/+}$ BMDMs in each assay was set to 100% (mean ± SEM from four independent assays with BMDMs from two mice in each assay; total *n* = 8/8). Norm., normalized; rel., relative. **(F)** Schematic representation of in vivo pneumonia model. The lungs of *Regnase-3*$^{fl/fl}$ +LysM-Cre and *Regnase-3*$^{fl/fl}$ littermate controls were inoculated with MRSA. 24 h after inoculation, organs were withdrawn to determine CFUs in lung tissue and to perform immunohistochemical analysis. **(G)** In vivo pneumonia model. CFUs in *Regnase-3*$^{fl/fl}$ +LysM-Cre and *Regnase-3*$^{fl/fl}$ littermate controls. **(H)** Immunofluorescence stains of lungs from pneumonia model. Lung sections were stained for *S. aureus* (green) and macrophages with anti-CD68 (red) and counterstained for nuclei with DAPI (blue). Images were acquired by confocal microscopy. Representative images are shown. Bars: 50 µm (left); 25 µm (right). Data are represented as mean ± SEM and were compared by Mann–Whitney *U* test. ns, not significant.
